# A blocking monoclonal antibody reveals dimerization of intracellular domains of ALK2 associated with genetic disorders

**DOI:** 10.1038/s41467-023-38746-5

**Published:** 2023-05-25

**Authors:** Takenobu Katagiri, Sho Tsukamoto, Mai Kuratani, Shinnosuke Tsuji, Kensuke Nakamura, Satoshi Ohte, Yoshiro Kawaguchi, Kiyosumi Takaishi

**Affiliations:** 1 Division of Biomedical Sciences, Research Center for Genomic Medicine, Saitama Medical University, 1397-1 Yamane, Hidaka-shi, Saitama 350-1241 Japan; 2 Project of Clinical and Basic Research for FOP, Saitama Medical University, 1397-1 Yamane, Hidaka-shi, Saitama 350-1241 Japan; 3 Specialty Medicine Research Laboratories I, R&D Division, Daiichi Sankyo Co., Ltd., 1-2-58 Hiromachi, Shinagawa-ku, Tokyo, 140-8710 Japan; 4 Modality Research Laboratories, Biologics Division, Daiichi Sankyo Co., Ltd., 1-2-58 Hiromachi, Shinagawa-ku, Tokyo, 140-8710 Japan; 5 Present Address: Graduate School of Pharmaceutical Sciences, Kitasato University, 5-9-1 Shirokane, Minato-ku, Tokyo, 108-8641 Japan

**Keywords:** Translational research, Molecular medicine, Growth factor signalling

## Abstract

Mutations in activin receptor-like kinase 2 (ALK2) can cause the pathological osteogenic signaling seen in some patients with fibrodysplasia ossificans progressiva and other conditions such as diffuse intrinsic pontine glioma. Here, we report that intracellular domain of wild-type ALK2 readily dimerizes in response to BMP7 binding to drive osteogenic signaling. This osteogenic signaling is pathologically triggered by heterotetramers of type II receptor kinases and ALK2 mutant forms, which form intracellular domain dimers in response to activin A binding. We develop a blocking monoclonal antibody, Rm0443, that can suppress ALK2 signaling. We solve the crystal structure of the ALK2 extracellular domain complex with a Fab fragment of Rm0443 and show that Rm0443 induces dimerization of ALK2 extracellular domains in a back-to-back orientation on the cell membrane by binding the residues H64 and F63 on opposite faces of the ligand-binding site. Rm0443 could prevent heterotopic ossification in a mouse model of fibrodysplasia ossificans progressiva that carries the human R206H pathogenic mutant.

## Abstract (web summary)

Mutant forms of the transmembrane receptor activin receptor-like kinase 2 are associated with different genetic diseases. Here, the authors report that disease-associated forms of this receptor cause intracellular domains to dimerize in response to ligand binding of extracellular domains.

## Introduction

Activin receptor-like kinase 2 (ALK2) is a transmembrane kinase receptor that activates osteogenic signaling in response to binding dimeric ligands, such as bone morphogenetic proteins (BMPs), of the transforming growth factor-β (TGF-β) family^1,2^. ALK2 belongs to the type I receptor family (ALK1 through ALK7) and transmits signals by phosphorylating transcription factors, such as Smad1, Smad5 and Smad9 (also known as Smad8)^3–5^. ALK2 and other type I receptors are translated as inactive kinases but activated by five types of type II receptor kinases as substrates in response to ligand binding. A domain rich in glycine and serine (GS) (amino acids 178–207) in the cytoplasmic juxtamembrane region of ALK2 is conserved among type I receptors and is the site phosphorylated by type II receptors. Genetic substitution of glutamine residue 207 in the GS domain to aspartic acid (Q207D) constitutively activates the ALK2 kinase without ligand stimulation^6,7^. Mutated Q207D was further enhanced by co-expression of BMP type II receptors, such as ActR-IIB and BMPR-II, even in the context of deficient kinase activity^8–10^.

Natural gain-of-function mutations of ALK2 were found in patients with genetic disorders^11^. Fibrodysplasia ossificans progressiva (FOP; OMIM #135100) is a rare autosomal dominant disorder characterized by progressive heterotopic ossification of soft tissues^12–14^. There are no approved drugs to prevent heterotopic ossification in FOP other than Palovarotene (selective retinoic acid receptor γ agonist), which is approved only in Canada. Substitution of the arginine residue at position 206 to histidine (R206H) in the GS domain of ALK2 was found in patients with familial and sporadic FOP^15^. Twelve additional mutations in the GS and kinase domains of ALK2 were found in patients with FOP^16–25^. Mutations found in FOP are clustered in the intracellular domain of ALK2^26^. Of the thirteen ALK2 mutations involved in FOP, including R206H, six mutations and an additional mutation, G328V, were identified in patients with, diffuse intrinsic pontine glioma (DIPG), a type of severe pediatric brain tumor^27–30^. Furthermore, a unique mutation, K400E, was identified in ALK2 in a patient with diffuse idiopathic skeletal hyperostosis (DISH; OMIM #106400), characterized by calcification of the spinal ligaments and enthesis without heterotopic ossification of the soft tissues, but not FOP^31^. Mutated ALK2 in FOP and DIPG transmits greater signaling in response to osteogenic ligands, including BMP7, than wild-type ALK2^32–34^. Mutated ALK2 in FOP, DIPG, and DISH is hyper-phosphorylated by BMP type II receptors, such as ActR-IIB and BMPR-II^8,35^. Moreover, activin A, a non-osteogenic ligand in the TGF-β family, induced heterotopic ossification in a mouse model of FOP carrying R206H mutant ALK2, and this effect was blocked by a neutralizing antibody for activin A^32,33^. However, the DISH-associated mutant K400E ALK2 showed a higher response to BMP7 but not activin A^35^. Activin A activates aberrant signaling through the R206H mutant ALK2, but not wild-type ALK2, but the molecular mechanisms have not been clarified^36^. Here, we investigated the molecular mechanisms of receptor assembling involved in the mutated ALK2 signaling pathway and examined the potential of a blocking antibody that inhibits ligand-dependent signal induction through mutated ALK2 as an effective drug for FOP.

## Results

### The isolated rat monoclonal antibody Rm0443 selectively inhibits ALK2 signaling and prevents ligand-induced heterotopic ossification

**Fig. 1:**
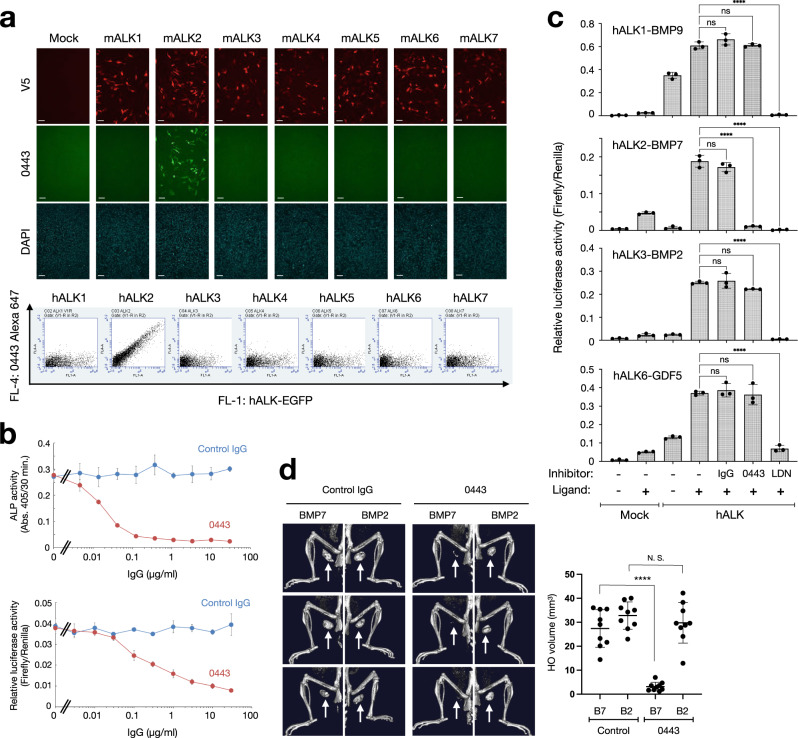
Development of rat monoclonal antibodies against ALK2. **a** Specific binding of Rm0443 to ALK2 shown by immunocytochemical (upper panels) and flow cytometry (lower panels) analyses. C2C12 cells were individually transfected with V5-tagged mouse ALK1 through ALK7. The cells were stained with antibodies against clone V5005 (for the V5-tag) and Rm0443 and then visualized with Alexa Fluor 594- and Alexa Fluor 488-conjugated secondary antibodies, respectively. The scale bars represent 50 μm. HEK293A cells expressing one of the EGFP-tagged human ALK1 to ALK7 constructs were analyzed with fluorescent dye-conjugated Rm0443. Two independent experiments were performed for both analyses. **b** Inhibitory activity of Rm0443 against ALP activity (upper panel) and BMP-specific reporter activity (lower panel) in C2C12 cells. C2C12 cells were incubated with 300 ng/ml BMP7 and increasing concentrations of control IgG or Rm0443. ALP activities in the cultures were determined on day 3. The cells were transfected with reporter plasmids. The cells were incubated overnight with 300 ng/ml BMP7 and increasing concentrations of control IgG or Rm0443. IC50 values of Rm0443 for ALP and luciferase reporter were 0.017 and 0.216 μg/ml, respectively. The lowest concentration that gave a dose response represents the absence of antibodies. (*n* = 3, biologically independent samples). Data were expressed as the mean ± S.D. Source data are provided as a Source data file. **c** Effects of Rm0443 and a pan-BMP receptor kinase inhibitor, LDN-193189, on BMP type I receptors in vitro. HEK293A cells were transfected with reporter plasmids containing ALK1, ALK2, ALK3, or ALK6. The cells were incubated overnight with 0.5 ng/ml BMP9, 10 ng/ml BMP7, 10 ng/ml BMP2, or 10 ng/ml GDF5 in the presence of 10 μg/ml antibody (control IgG or Rm0443) or 1.0 μM LDN-193189 (*n* = 3, biologically independent samples). Data were expressed as the mean ± S.D. Source data are provided as a Source data file. **d** Effects of Rm0443 on BMP-induced heterotopic ossification in vivo. Male mice at 9-week-old were transplanted with collagen pellets containing 2 μg of BMP2 (right legs) and 5 μg of BMP7 (left legs) and injected subcutaneously 10 mg/kg/week of control IgG or Rm0443. Heterotopic ossification was visualized by μCT on day 14 (left panels) and quantified (right panel). Data were expressed as the mean ± S.D. (*n* = 9 for control IgG: *n* = 9 for Rm0443). Source data are provided as a Source data file. *P* values are calculated using unpaired one-way ANOVA and indicated significant if ****P < 0.0001.

We developed a hybridoma cell line that secretes a rat anti-ALK2 antibody clone #0443 (Rm0443). Rm0443 was found to specifically bind mouse and human ALK2 among ALK1 through ALK7 with KD values of 5.1 and 5.6 nM, respectively (Fig. 1a and Supplementary Fig. 1a). Rm0443 inhibited alkaline phosphatase (ALP) activity and BMP-specific luciferase reporter activity induced by 300 ng/ml BMP7 in murine C2C12 myoblasts, which express endogenous ALK2 (Fig. 1b), and HEK293A cells overexpressing mouse or human ALK2 (Supplementary Fig. 1b). Rm0443 recognized the ~60 k monomeric, ~150 k dimeric and >150 k multimeric forms of mouse and human ALK2 in western blot analysis under non-reducing conditions but not under reducing conditions (Supplementary Fig. 1c, d). In contrast to a pan-BMP receptor kinase inhibitor, LDN-193189, Rm0443 specifically inhibited reporter activity through ALK2 among BMP type I receptors (Fig. 1c). Rm0443 administration significantly reduced the induction of heterotopic endochondral ossification by BMP7, but not BMP2, which is known to bind ALK3 rather than ALK2, in the same mice (Fig. 1d and Supplementary Fig. 1e).

### Rm0443 binds ALK2 through the residue H64 and inhibits ALK2

**Fig. 2:**
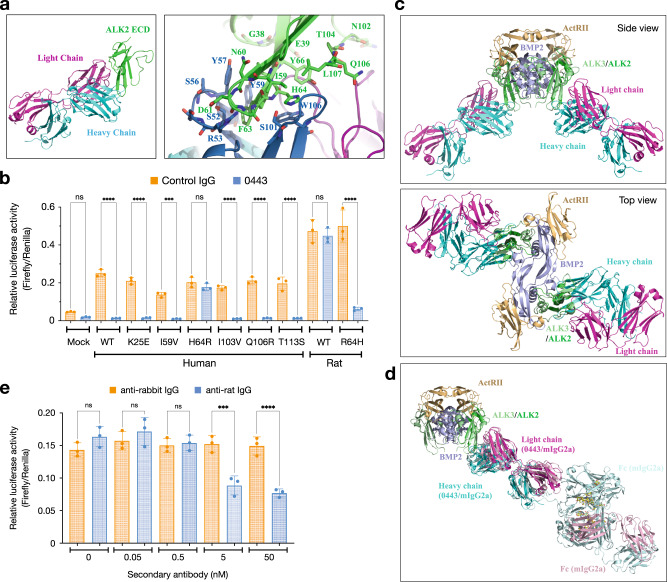
Rm0443 inhibits ALK2 through binding the residue H64. **a** Crystal structures of a complex consisting of a Rm0443 Fab fragment and the human ALK2 ECD. Ribbon models of the interface between ALK2 (green) and the heavy (cyan) and light (magenta) chains of Rm0443. Amino acids and the position of each residue are indicated in the figure. The right panel provides a close-up view of the left panel. **b** The H64 residue in ALK2 is essential for the inhibitory effect of Rm0443. Amino acids in human and rat ALK2 were substituted as indicated in the figure. HEK293A cells were transfected with human and rat ALK2 expression vectors with reporter plasmids. The cells were treated overnight with 10 ng/ml BMP7 and 3 μg/ml control IgG or Rm0443 (*n* = 3, biologically independent samples). Data were expressed as the mean ± S.D. Source data are provided as a Source data file. **c** Side and top views of the ALK2-Rm0443 complex superimposed on each of the ALK3 proteins in the ALK3-ActR-II-BMP2 signaling complex (PDB ID: 2GOO). The RMSD of the main chain atoms of β3, β4 and β5 of ALK3 (PDB ID: 2GOO) and the corresponding atoms of ALK2 was 0.54 Å, although the β4-β5 loop structure of ligand-free ALK2 was as dissimilar to that of ALK3 (PDB ID: 2GOO) as that of ligand-free ALK3 (PDB ID: 3NH7) possibly owing to the crystal contact observed in that region of ALK2. The signaling complex is colored as follows: pale green (ALK3/ALK2), pale blue (BMP2), and pale orange (ActRII). The Rm0443 Fab fragment (cyan and magenta for the heavy and light chains, respectively) binds the peripheral surface of ALK2, which may be in complex with the ligand and type II receptor ECD, although the simultaneous binding of two Fab fragments on the cell surface is implausible. **d** Structural modeling of Rm0443 Fab-ALK2 and mouse IgG2a (PDB ID: 1IGT) with the ALK3-ActR-II-BMP2 complex (PDB ID: 2GOO). A single Fab or IgG2a can bind to one of the two ALK ECDs in the ligand-bound complex. **e** The Rm0443 Fab fragment inhibited signaling in the presence of a secondary antibody. HEK293A cells were transfected with human ALK2(WT) and reporter plasmids. The cells were treated with 10 ng/ml BMP7 and 200 nM Rm0443 Fab fragment with increasing concentrations of secondary antibody against rabbit (orange column) or rat (blue columns) (*n* = 3, biologically independent samples). Source data are provided as a Source data file. *P* values are calculated using unpaired one-way ANOVA and indicated significant if ***P < 0.001 or ****P < 0.0001.

We analyzed the crystal structure of ALK2 and the binding epitopes for Rm0443 by crystallography analysis at 2.6 Å resolution. Rm0443 mainly interacts with the β3 and β4 of ALK2 (Fig. 2a). The consecutive ALK2 residues F63 and H64 that are distinct among ALK receptors (Supplementary Fig. 2a) show extensive interactions with complementarily determining regions (CDRs) of the heavy chain of Rm0443 (Fig. 2a). The side chain of ALK2 H64 stacks on the W106 and forms hydrogen-bond with S101 of the heavy chain. The side chain of ALK2 F63 is buried between main chain and hydrophobic side chains of the heavy chain that include Y57 which may also form hydrogen-bond with side chain of ALK2 N60. The side chain of adjacent ALK2 D61 forms hydrogen-bond with the main chain of S54 of the heavy chain, and ALK2 D61 main chain hydrogen-bonds with the main chain of R53 of the heavy chain. The ALK2 residue Y66 that is also distinct among ALK receptors, makes hydrophobic interactions with main chain of the light chain CDRs. The residues in ALK2 that interact with Rm0443 are highly conserved among the animal species humans, monkeys, horses, dogs, beavers, gerbils, and mice (Supplementary Fig. 2b). Rm0443 did not inhibit BMP7-induced signaling via elephant, rat, or chicken ALK2, which contain unique residues in the binding epitopes (Supplementary Fig. 3a, b). The extracellular domain of rat ALK2 contains six unique residues. The substitution of H64 in human ALK2 to arginine (H64R) blocked Rm0443-mediated inhibition, and the substitution R64H in rat ALK2 had the opposite effect (Fig. 2b). The elephant and chicken ALK2 proteins also became sensitive to Rm0443 with the Y63F and A55F/K56H (corresponding to F63 and H64, respectively, in human ALK2) substitutions, respectively (Supplementary Fig. 3b). Rm0443 bound sensitive ALK2 but did not bind rat, chicken, or human ALK2 (H64R) (Supplementary Fig. 3c). These results indicate that H64 in ALK2 is essential for the binding of Rm0443 to and its inhibition of ALK2.

Pretreatment of cells with BMP7 or activin A did not reduce the capacity of Rm0443 to bind ALK2 (Supplementary Fig. 3d), suggesting that Rm0443 does not directly compete with the ligand for ALK2 bind. Superimposing our structure on the crystal structure of a published ternary complex consisting of a BMP2 dimer, ALK3 and the ActR-II extracellular domains (RCSB PDB ID: 2GOO) suggests that Rm0443 Fab fragments bind the outside of the ALK2-ligand complex and flanked the ternary complex (Fig. 2c, d). While Weber et al.^37^ estimates that the ligand-dimer complex resides approximately 20 Å from the cell membrane surface, the simultaneous binding of two Rm0443 Fab fragments would require that distance to be approximately 60 Å which is implausible. It is, however, possible for a single Fab to lie parallel to the membrane and bind to slightly tilted signaling complex, precluding the binding of another Fab to the other ALK2 in the complex. Accordingly, the Rm0443 Fab fragment did not inhibit BMP signaling (Supplementary Fig. 3e); however, the addition of an anti-rat secondary antibody restored its inhibitory capacity (Fig. 2e and Supplementary Fig. 3f). We could model an ALK2-bound Rm0443 through a single arm of mouse IgG2a (PDB ID: 1IGT). Among divalently bound antibody conformations, it is reasonable to assume that antibody with Fab-arms facing toward the membrane would be in far less constraint than that with Fab-arms parallel to the membrane in T-shaped forms observed in a solution structural study by Tian et al.^38^. Taken together, these results suggest that Rm0443 inhibits ligand-induced signaling by forming a back-to-back dimer of ALK2 on the cell membrane.

### Mutated ALK2 form substantial intracellular dimers in response to ligand stimulation

**Fig. 3:**
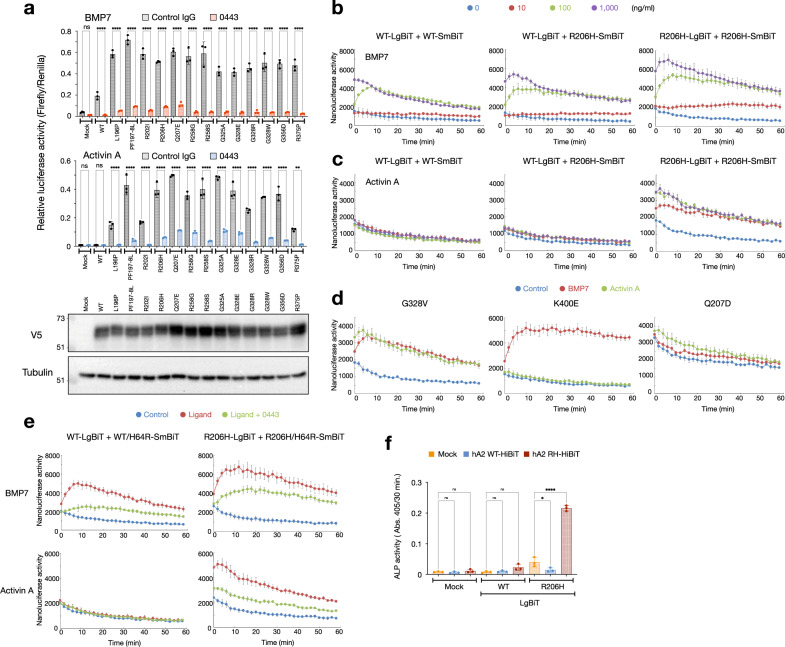
Mutated ALK2 proteins associated with genetic disorders form substantial amounts of ALK2 ICD dimers in response to ligand stimulation. **a** Rm0443 inhibits BMP signaling through mutant ALK2 proteins associated with FOP in response to BMP7 and activin A. HEK293A cells were transfected with wild-type ALK2 or mutant ALK2 associated with FOP with reporter plasmids. The cells were treated with 10 ng/ml BMP7 (upper panel) and 10 ng/ml activin A (middle panel) with 3 μg/ml control IgG or Rm0443 (*n* = 3, biologically independent samples). Data were expressed as the mean ± S.D. The expression levels of ALK2 were determined by western blot using an antibody against V5-tag (bottom panels). Source data are provided as a Source data file. **b**, **c** Formation of dimers of ALK2 ICDs in response to BMP7 (**b**) and activin A (**c**). HEK293A cells were transfected with ALK2-LgBiT and ALK2-SmBiT containing wild-type and R206H ALK2 with FLAG (unrelated)-tagged ActR-IIB as indicated and stimulated with 0 (blue), 10 (red), 100 (green), or 1000 ng/ml (purple) BMP7 (**b**) and activin A (**c**). Nanoluciferase activity was determined every 2 min up to 1 h after stimulation (*n* = 3, biologically independent samples). Data were expressed as the mean ± S.D. Source data are provided as a Source data file. **d** Dimer formation of G328V, K400E, and Q207D ALK2 ICDs in response to BMP7 and activin A stimulation. HEK293A cells were transfected with ALK2-LgBiT and ALK2-SmBiT containing each mutated ALK2 protein with FLAG (unrelated)-tagged ActR-IIB as indicated and stimulated without (control, blue) or with 100 ng/ml BMP7 (red) and 100 ng/ml activin A (green) (*n* = 3, biologically independent samples). Data were expressed as the mean ± S.D. Source data are provided as a Source data file. **e** Rm0443 suppressed the dimer formation ALK2 ICDs in response to ligand stimulation. HEK293A cells were transfected with ALK2-LgBiT and ALK2-SmBiT or ALK2(H64R)-SmBiT consisting of wild-type ALK2 (left panels) and R206H ALK2 (right panels) with FLAG (unrelated)-tagged ActR-IIB as indicated and stimulated with 100 ng/ml BMP7 (upper panels) and 100 ng/ml activin A (lower panels) in the presence of 10 μg/ml Rm0443 (*n* = 3, biologically independent samples). Data were expressed as the mean ± S.D. Source data are provided as a Source data file. **f** The forced interaction of ALK2(R206H) ICDs induced ALP activity in the absence of ligand. C2C12 cells were transfected with a mock vector, ALK2(WT)-LgBiT, or ALK2(R206H)-LgBiT within the pcDEF3 expression vector. ALP activity was determined on day 6 (*n* = 3, biologically independent samples). Data were expressed as the mean ± S.D. Source data are provided as a Source data file. *P* values are calculated using unpaired one-way ANOVA and indicated significant if *P < 0.05, **P < 0.01, or ****P < 0.0001.

The activation of BMP signaling by BMP7 and activin A was suppressed by Rm0443 in thirteen forms of mutated ALK2 associated with FOP (Fig. 3a). We examined the real-time interactions of ALK2 intracellular domain (ICD) in living cells using NanoBiT, a highly sensitive nanoluciferase reporter assay for protein-protein interactions, in response to ligand stimulation in the presence of a type II receptor, ActR-IIB (Supplementary Fig. 4a)^39^. Stimulation with BMP7 dose-dependently increased the interaction of wild-type and R206H ALK2 ICDs; 100 ng/ml BMP7 increased the interaction with peaks from minutes 6–10, and 1000 ng/ml BMP7 increased the interaction with peaks within 6 min (Fig. 3b). Activin A did not induce a complex containing wild-type ALK2 ICD even at 1000 ng/ml but increased the interaction of R206H ALK2 ICDs within 2 min at 10 ng/ml and greater (Fig. 3c). Myostatin did not induce the formation of homodimer and heterodimer of both WT and R206H ALK2 ICDs (Supplementary Fig. 4b). Other mutated ALK2 proteins involved in the genetic disorders FOP and DIPG (Q207E, R258G, G328E, and G356D), unique to FOP (R258S and G325A), and unique to DIPG (G328V) also formed the ICD complexes in a few minutes in response to both BMP7 and activin A (Fig. 3d and Supplementary Fig. 4c). In contrast, dimerization of K400E ALK2 ICDs, unique to DISH, was maintained at a high level with BMP7 but not activin A (Fig. 3d). The genetically engineered constitutively active mutant Q207D ALK2 formed the largest amount of the complex without ligand stimulation and showed minimal ligand dependency (Fig. 3d). The BMP7-, activin A-, and myostatin-induced ICD dimer formation of the wild-type, R206H, G328V, K400E, and Q207D ALK2 occurred in parallel with signal activation (Supplementary Fig. 4d). These findings suggest that mutated ALK2 associated with FOP, DIPG, and DISH form substantial amounts of ICD dimers in response to active ligands, including activin A. Rm0443 reduced the heteromeric dimer formation of ALK2 ICDs between H64R and the wild-type induced by BMP7 and activin A (Fig. 3e), suggesting that Rm0443 suppresses the ligand-induced dimer formation of ICDs. Transfection of ALK2(R206H)-LgBiT with a mock vector induced weak ALP activity in C2C12 cells, which was increased by co-expression with ALK2(R206H)-HiBiT, which has an affinity 2.7 ×10^6^-fold higher for LgBiT than for SmBiT and autonomously forms a dimer with LgBiT (Fig. 3f)^39,40^. The basal ALP activity of ALK2(R206H)-LgBiT was reduced by co-expression of ALK2(WT)-HiBiT (Fig. 3f), suggesting that a heterodimer containing ALK2(WT) ICD is inactive.

### Type II receptors are indispensable for the formation of ALK2 ICD dimers

**Fig. 4:**
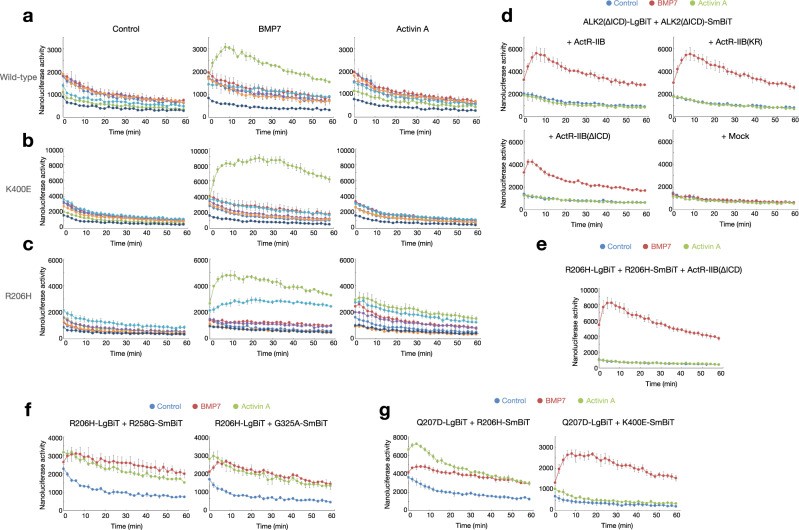
Type II receptors assemble the dimer formation of ALK2 ICDs in response to ligand stimulation. **a**–**c** Type II receptors assemble the dimer formation of ALK2 ICDs. HEK293A cells were transfected with ALK2-LgBiT and ALK2-SmBiT containing wild-type (**a**), K400E (**b**), and R206H (**c**) ALK2 with FLAG (unrelated)-tagged type II receptors as indicated. The cells were stimulated without (left) and with 100 ng/ml BMP7 (middle) and 100 ng/ml activin A (right) (n = 3, biologically independent samples). Data were expressed as the mean ± S.D. Source data are provided as a Source data file. **d** Dimer formation of ALK2(ΔICD) in the presence of full-length and mutant ActR-IIB. HEK293A cells were transfected with ALK2(ΔICD)-LgBiT and ALK2(ΔICD)-SmBiT with FLAG (unrelated)-tagged ActR-IIB, kinase activity-deficient ActR-IIB(KR) or ActR-IIB(ΔICD). The cells were stimulated without (blue) and with 100 ng/ml BMP7 (red) and 100 ng/ml activin A (green) (*n* = 3, biologically independent samples). Data were expressed as the mean ± S.D. Source data are provided as a Source data file. **e** Dimer formation of ALK2(R206Η) ICDs in the presence of ActR-IIB(ΔICD). HEK293A cells were transfected with ALK2(R206H)-LgBiT and ALK2(R206H)-SmBiT with FLAG (unrelated)-tagged ActR-IIB(ΔICD). The cells were stimulated without (control, blue) and with 100 ng/ml BMP7 (red) and 100 ng/ml activin A (green) (*n* = 3, biologically independent samples). Data were expressed as the mean ± S.D. Source data are provided as a Source data file. **f**, **g** The interaction of mutated ALK2 proteins with each other. HEK293A cells were transfected with ALK2(R206H)-LgBiT (**f**) or ALK2(Q207D)-LgBiT (**g**) with ALK2-SmBiT containing R258G, G325A (**f**), R206H, and K400E (**g**) ALK2 as indicated with FLAG (unrelated)-tagged ActR-IIB. The cells were stimulated without (control, blue) and with 100 ng/ml BMP7 (red) and 100 ng/ml activin A (green) (*n* = 3, biologically independent samples). Data were expressed as the mean ± S.D. Source data are provided as a Source data file.

ActR-IIB induced ICD homodimers of wild-type, K400E, R206H and Q207D ALK2 upon stimulation with ligands (Fig. 4a–c and Supplementary Fig. 5a, b). Although ActR-IIB showed the most potent induction, other type II receptors, including ActR-IIA and BMPR-II, also increased the ICD dimers of R206H and Q207D ALK2 in response to BMP7 and activin A (Fig. 4c and Supplementary Fig. 5b). Activin A, but not BMP7, rapidly induced the dimer formation of ActR-IIB ICDs within 4 min after stimulation (Supplementary Fig. 5c). Both BMP7 and activin A minimally increased the interaction between ALK2 ICD and ActR-IIB ICD (Supplementary Fig. 5d). To examine the role of the ALK2 ICDs in their dimer formation, we constructed ALK2(ΔICD), which was truncated to remove most of the ICD. BMP7 still induced the dimer formation of ALK2(ΔICD) in the presence of ActR-IIB, even the forms deficient in kinase activity (KR) or ΔICD (Fig. 4d). In contrast, activin A did not induce ALK2(ΔICD) dimer formation even in the presence of WT ActR-IIB (Fig. 4d), suggesting that the interaction between ALK2 and ActR-IIB ICDs is indispensable. This hypothesis was supported by the dimer formation capacity of R206H ALK2 ICDs in the presence of ActR-IIB(ΔICD) in response to BMP7 and activin A (Fig. 4e). Furthermore, both R206H and Q207D ALK2 formed ICD heterodimers with other mutated ALK2 constructs, such as R258G and G325A, but not K400E, in response to activin A (Fig. 4f, g), suggesting that the mutated ALK2 ICDs involved in FOP and DIPG, but not DISH, have acquired the capacity to interact with each other in the presence of activin A.

### Rm0443 enhanced mouse ALK2(R206H) but inhibited human ALK2(R206H) due to the difference in amino acids at position 330

**Fig. 5:**
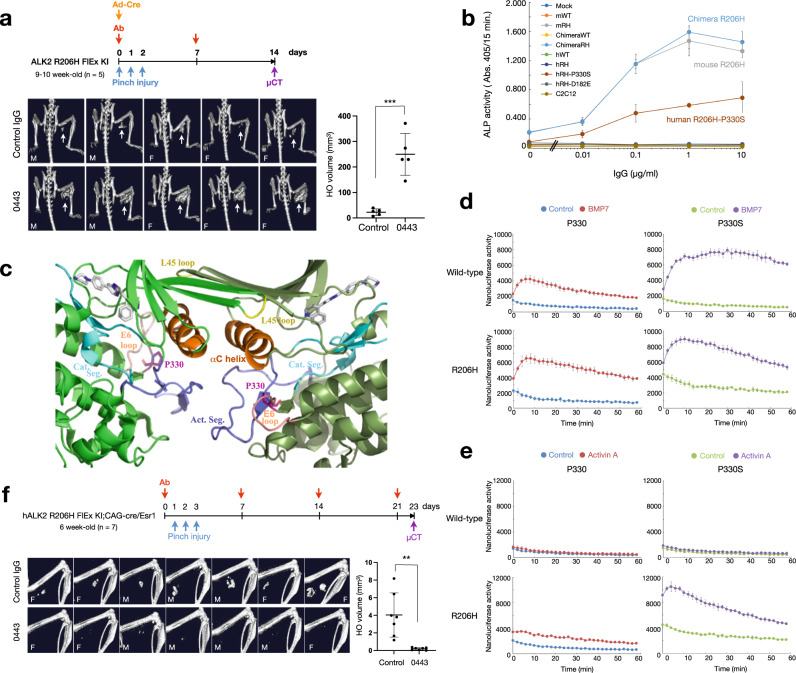
Rm0443 enhanced heterotopic ossification in a mouse model of mouse ALK2(R206H) but inhibited human ALK2(R206H). **a** Rm0443 enhanced heterotopic ossification in mice with locally induced mouse Alk2(R206H). (upper panel) Schematic representation of the experimental protocol. Mouse Alk2(R206H) FlEx KI mice were injected with Cre-expressing adenovirus in skeletal muscle to locally induce ALK2(R206H), after which they were subcutaneously injected with 10 mg/kg/week control IgG or Rm0443. (left panels) μCT images of mice treated with control IgG (upper panels) and Rm0443 (lower panels) on day 14. Arrows indicated heterotopic ossification. M (male) or F (female) indicated gender. (right panel) Quantified volumes of heterotopic ossification (arrows) in mice treated with control IgG and Rm0443. Data are expressed as the mean ± S.D. (*n* = 5 for control IgG; *n* = 5 for Rm0443). Source data are provided as a Source data file. **b** Rm0443 induced ALP activity in C2C12 cells expressing mouse ALK2(R206H) and human ALK2(R206H-P330S). C2C12 cells stably expressed human ALK2(WT), human ALK2(R206H), mouse ALK2(WT), mouse ALK2(R206H), chimeric ALK2(WT), chimeric ALK2(R206H), human ALK2(R206H-P330S), or human ALK2(R206H-D182E) and cultured with increasing concentrations of Rm0443. ALP activity was determined on day 6. The lowest concentration on the dose response curve represents the absence of antibody (*n* = 3, biologically independent samples). Data were expressed as the mean ± S.D. Source data are provided as a Source data file. **c** A ribbon model of a dimer of human ALK2 kinase domains (RCSB PDB ID: 3Q4U). P330 (magenta) residues are in the E6 loops (pink) and connected to the catalytic segments (light blue). The active segments (purple) and αC helixes (orange) are also shown. **d**, **e** The residue S330 increased dimer formation of an ALK2 ICDs in response to ligand stimulation. HEK293A cells were transfected with human ALK2-LgBiT and human ALK2-SmBiT with the parent residue (P330, left panels) or P330S mutation (right panels) consisting of wild-type ALK2 (upper panels) and R206H ALK2 (lower panels) with FLAG (unrelated)-tagged ActR-IIB as indicated. The cells were stimulated with 100 ng/ml BMP7 (**d**) and 100 ng/ml activin A (**e**). Reporter activity was determined every 2 min up to 1 h after stimulation (*n* = 3, biologically independent samples). Data were expressed as the mean ± S.D. Source data are provided as a Source data file. **f** Rm0443 suppressed heterotopic ossification in CAG-cre/Esr1;hALK2(R206H) FlEx KI mice. (upper panel) Schematic representation of the experimental protocol. (left panels) μCT images of mice subcutaneously injected with 10 mg/kg/week control IgG (upper panel) and Rm0443 (lower panel) on day 23. M (male) or F (female) indicated gender. (right panels) Quantified volumes of heterotopic ossification in mice treated with control IgG and Rm0443. Data are expressed as the mean ± S.D. (*n* = 7). Source data are provided as a Source data file. *P* values are calculated using unpaired one-way ANOVA and indicated significant if **P < 0.01 or ***P < 0.001.

Finally, to examine the potential of Rm0443 as a therapeutic antibody for FOP patients in a relevant animal model, we established a mouse model of FOP [mAlk2(R206H) FlEx KI], in which mouse Alk2(R206H) was knocked-in to the endogenous Alk2 locus and its expression was induced by Cre-dependent recombination (Supplementary Fig. 6a). Unexpectedly, heterotopic ossification induced by the local injection of Cre-expressing adenovirus under the control of cytomegalovirus promoter and pinch injury was further increased by Rm0443 administration (Fig. 5a).

In order to elucidate the mechanisms underlying the Rm0443-mediated enhancement of HO in the mouse models, we examined if Rm0443 can induce the signal activation of mouse ALK2(R206H) using C2C12 myoblasts stably expressing human or mouse ALK2. Rm0443 dose-dependently increased ALP activity in cells transfected with mouse ALK2(R206H) and a chimeric ALK2(R206H) protein containing the extracellular domain from human ALK2 and the intracellular domain from mouse ALK2 with R206H ALK2, but not others, including human ALK2(R206H) (Fig. 5b), indicating that Rm0443 enhanced the signaling cascade related to only ALK2(R206H) with the mouse intracellular sequence in the absence of ligand stimulation. The mouse intracellular domain of ALK2 contains two unique residues at E182 and S330, which correspond to D182 and P330, respectively, in human ALK2 (Supplementary Fig. 6b). Rm0443 induced ALP activity in C2C12 cells expressing human ALK2(R206H) carrying P330S but not D182E (Fig. 5b). The serine residue at position 330 in ALK2 is evolutionally conserved in only the subfamily of rodents that includes rats, gerbils, and mice, and Rm0443 enhanced activity of rat ALK2(R64H/R206H) and gerbil ALK2(R206H) (Supplementary Fig. 6b, c).

The P330 residue in human ALK2 resides in the E6 loop and is connected to the catalytic segment by a short strand of three amino acids, as shown in the published crystal structure (RCSB PDB ID: 3Q4U)^41^ (Fig. 5c). P330 is also located between the activation segment and the αC helix, which act as “steric blockers” of TβR-I when ALK2 is in an inactive conformation^42^. Substitution of P330 in human ALK2 with S330 increased the ICD dimers of both wild-type and R206H ALK2 in response to BMP7 and activin A stimulation (Fig. 5d, e). Further substitution analysis at position 330 indicated that proline and serine are not specific for suppression and enhancement induced by Rm0443 (Supplementary Fig. 6d). In addition, Rm0443 did not show any enhancement effect on thirteen forms of FOP-associated mutant human ALK2, rather suppressed their activity in vitro (Supplementary Fig. 6e). To further validate the potential of Rm0443 as a therapy for FOP patients, we established another mouse model of FOP in which the allele of inducible human ALK2(R206H) was knocked in [hALK2(R206H) FlEx KI;CAG-cre/Esr1]. Consistent with our in vitro data, the administration of Rm0443 significantly suppressed heterotopic ossification in this mouse model (Fig. 5f).

## Discussion

In this study, we identified a blocking antibody, Rm0443, that inhibited ALK2 signaling in vitro and in vivo, and we solved the crystal structures of the ALK2 ECD complex with a Fab fragment of Rm0443. Our findings suggest that Rm0443 can induce a unique dimer of ALK2 ECD in a back-to-back orientation on the cell membrane by binding the residues H64 and F63 on opposite faces of the ligand-binding surface (Supplementary Fig. 7). This unique conformation of the ALK2 ECD-Rm0443 complex may be important for understanding the molecular mechanisms of signal activation and inhibition on the cell membrane. During the review of this manuscript, antibodies against ALK2 were reported by others^43,44^. These reports showed that anti-ALK2 antibodies exacerbated heterotopic ossification in mouse models of FOP expressing mouse ALK2(R206H), which were consistent with our results. In contrast to our Rm0443, the Fab fragments of their antibodies still inhibited ALK2 signaling^43^, suggesting that those antibodies bind to different epitopes of ALK2 from that of Rm0443, which binds to the outside of the ALK2 ECD-ligand complex. Rm0443 would be useful not only for basic research but also for the development of a therapeutic drug for genetic disorders associated with mutated ALK2. Because Rm0443 is a rat antibody, we developed humanized antibodies for clinical use in treating genetic disorders associated with ALK2. One of the antibodies, DS-6016, was started in the phase I trial for FOP with healthy volunteers in Japan.

**Fig. 6:**
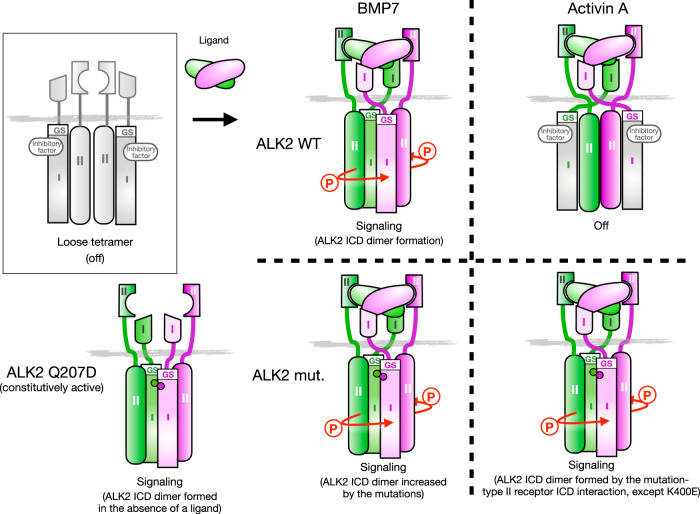
A schematic model of the ALK2 activation through ICD dimer formation induced by BMP7 and activin A. In the absence of ligands, ALK2 and type II receptor ICDs form a “loose tetramer” on the cell membrane, in which each ALK2 ICDs are prevented from interacting with each other by a putative inhibitory factor to mask the dimer-forming surface. An active ligand (dimer) binds the loose tetramer and induces the formation of the tight tetramer on the cell membrane, consequently inducing the dimer formation of ALK2 ICDs. Genetic mutations in FOP and DIPG (circles) in the ALK2 ICDs release inhibitory factors and cause substantial amounts of ICD dimerization in response to ligand stimulation. Please see the Discussion for the details.

At least under experimental conditions, not only BMP7 but also activin A can activate pathological osteogenic signaling via mutated ALK2 associated with rare genetic disorders, such as FOP and DIPG^32,33^. In the present study, we identified a molecular mechanism underlying the rapid dimer formation of ALK2 ICDs for signaling in response to ligand stimulation (Fig. 6). This dimer formation of ALK2 ICDs is highly dependent on type II receptors, but the major domains of the type II receptors involved in the formation were different for each ligand. BMP7 induced the ALK2 dimer with dependence on the ActR-IIB ECDs and the transmembrane domain even though there is no direct interaction between type I and type II receptor ECDs^2,45,46^. In contrast, the ActR-IIB ICDs were indispensable for the mutated ALK2 dimer formation induced by activin A, suggesting that pathological signaling is activated through the interaction of type II receptor ICDs with ALK2 ICDs containing the mutation (Fig. 6). We detected the ICD dimers of ALK2/ActR-IIB, ActR-IIB/ActR-IIB and ALK2/ALK2 using NanoBiT, which suggested that these receptor ICDs are preformed as an inactive “loose tetramer” in the absence of ligands (Fig. 6). Recently, the preformed dimer between ALK2 and BMPR-II kinases was reported to be mediated through their C-terminal lobes^47^. This finding supports our hypothesis that ALK2 and type II receptors form a loose tetramer on the membrane before binding to a ligand. Because inactive ligands of WT ALK2, such activin A and myostatin, did not induce the dimer formation of ALK2 ICDs, it was suggested that there was an “inhibitory factor to mask the dimer-forming surface in ALK2 ICDs” under the inactive conditions (Fig. 6). Chaikuard et al.^26^ reported that FOP-associated mutations are clustered in the intracellular domain of ALK2 and break the inactive conformation of the kinase suppressed by the GS domain. The αC helix FKBP12 may act as an inhibitory factor for ALK2 that binds the GS domain^26,48,49^. However, in vitro sensitivity to FKBP12 and the clinical characteristics of a patient with FOP are inconsistent^18,34^, suggesting that another factor might prevent the dimerization of ALK2 ICDs under inactive conditions. Genetic mutations in ALK2 that are involved in FOP and DIPG may release the inhibitory factor from ALK2 ICDs and induce substantial amounts of ICD dimers in response to ligand stimulation. This hypothesis is further supported by the constitutively active Q207D ALK2, which formed the ICD dimer even in the absence of ligands (Fig. 6). Deletion of the wild-type Alk2 allele enhanced heterotopic ossification in a mouse model of FOP with Alk2(R206H) expression^50^. We found that the forced interaction of wild-type ALK2 and R206H mutant ALK2 failed to induce ALP activity in C2C12 cells. Such a dominant-negative ALK2 interaction may be another molecular mechanism by which Rm0443 inhibits ALK2 signaling.

Our mechanistic analysis revealed that the opposite effects of Rm0443 on human and mouse ALK2 resulted from the difference in a single residue at position 330. To the best of our knowledge, no other study has noted the difference between human and mouse ALK2 or the importance of the amino acid residue at position 330 of ALK2. The present data indicated that S330 in mouse ALK2 may disrupt the inhibitory conformation of the intracellular domain in the Rm0443-induced dimer. We found that ALK2 carrying S330 exhibited an increase in the interaction of the intracellular domains in response to ligand stimulation irrespective of the R206H mutation. It is possible that S330 is phosphorylated by a kinase in the cytoplasm, such as a type II receptor, due to substitution of the residue at 330 to the acidic residues aspartic acid and glutamic acid, as ALK2(R206H) showed higher activity in response to Rm0443. However, substitutions to other residues, including alanine, also enhanced activity in response to Rm0443, indicating that phosphorylation is not essential for the increase in activity induced by Rm0443. Additional studies will be needed to elucidate the physiological role of S330 in osteogenic signaling in rodent ALK2.

## Methods

All animal studies in this study were approved by the Institutional Animal Care and Use Committee and conducted according to the Saitama Medical University Animal Experimentation Regulations (#3133).

### Plasmids

Expression plasmids for mouse and human ALK1 through ALK7; type II receptors (ActR-IIA, ActR-IIB, BMPR-II short form, BMPR-II long form, AMHR-II and TβR-II); and monkey (accession #NM_001260761.1), elephant (accession #XM_010586301.1), horse (accession #XM_023622946.1), dog (accession #XM_549615.5), beaver (accession #XM_020167527.1), rat (accession #NM_024486.1), gerbil (accession #XM_021638758.1), and chicken (accession #NM_204560.1) ALK2 were constructed in the pcDEF3, pBit1.1-C[TK/LgBiT], and pBit2.1-C[TK/SmBiT] vectors (Promega, Madison, WI, USA) using a standard protocol as described previously (Supplementary Table 1). ALK2 mutants and type II receptors were created with a standard protocol as described previously (Supplementary Table 1). The DNA sequences were confirmed using an ABI3500 genetic analyzer (Applied Biosciences, Foster City, CA, USA).

### Development of Rm0443, a rat monoclonal antibody against ALK2

Hybridomas expressing monoclonal antibodies against ALK2 were established using a standard protocol (Integrale Co., Tokyo, Japan). In brief, female Wistar rats were immunized with 6x His-tagged mouse ALK2 ECD fused to human IgG Fc (Sino Biological, Inc., Beijing, China). Splenic cells were fused with P3U1 cells using 50% polyethylene glycol. The fused cells were cultured in RPMI 1640 medium containing 15% fetal bovine serum (FBS) and HAT supplement and were selected from the conditioned medium by ELISA using the antigen and human IgG Fc as a negative control. Antibodies from the selected clones were purified from the conditioned medium of serum-free Hybridoma-SFM (Thermo Fisher, Waltham, MA, USA) by affinity chromatography using a protein G column (GE Healthcare, Chicago, IL, USA) and the Profinia protein purification system (Bio-Rad Laboratories, Hercules, CA, USA). The Fab fragment of Rm0443 was prepared by papain digestion of Rm0443 and purified using protein A (Immuno-Biological Laboratories Co., Ltd., Gunma, Japan).

### Cell lines

Murine C2C12 cells were purchased from ATCC. Human embryonic kidney 293A (HEK293A) cells and FreeStyle 293F cells were obtained from Thermo Fisher Scientific. C2C12 and HEK293A cell lines were cultured in Dulbecco’s Modified Eagle Medium supplemented with 15% and 10% fetal bovine serum FBS, respectively. Cell lines were maintained at 37 °C in humidified CO2 (5%) incubators. FreeStyle 293F cell line was cultured in FreeStyle293 expression medium (Thermo Fisher Scientific) according to the manufacture’s protocol. All cell lines were tested negative for mycoplasma.

### Osteoblastic differentiation assay

C2C12 cells were used to examine the osteogenic signaling of ALK2 in vitro. ALP activity is a marker of osteoblastic differentiation of the cells^51,52^. C2C12 cells were cultured in 96-well plates with medium containing 15% FBS for 3 or 6 days. The ALP activity was measured by adding 100 μl of the ALP buffer containing a substrate (100 mM diethanolamine, 0.5 mM MgCl_2_ and 1 mg/ml p-nitrophenylphosphate) to each well. After incubation for 15 or 30 min at room temperature, the enzyme reaction was terminated by adding 50 μl of 3 M NaOH and absorbance was measured at 405 nm using Infinite F50 (Tecan, Männedorf, Switzerland). Data are expressed as the mean ± S.D.

### Luciferase reporter assay

BMP signaling via ALK1 through ALK7 was determined with the Dual-Glo luciferase assay (Promega) using Id1WT4F-luc for the Smad1 and Smad5 pathways^53–55^. phRL-SV40 (Promega) was used for normalization by Renilla activity. C2C12 cells and HEK293A cells inoculated in 96-well plates were transfected with Id1WT4F-luc, phRL-SV40 and a mixture of expression vectors for target proteins using Lipofectamine 2000 reagent (Thermo Fisher Scientific) following the manufacturer’s instructions. The culture medium containing the transfection reagents was changed to fresh OPTI-MEM (Thermo Fisher Scientific) after 2.5 h. The reporter activity was carried out using GENios (Tecan) and FLUOstar Omega (BMG LabTech, Aylesbury, UK). Data are expressed as the mean relative luciferase activity (Firefly/Renilla) ± S.D. The ligands used are summarized in Supplementary Table 2.

### Nanoluciferase assay for protein–protein interactions

NanoBiT (Promega) was applied to examine real-time protein-protein interactions in living cells. LgBiT and SmBiT were fused to the C-termini of two target proteins in each expression vector. HEK293A cells were transfected with a mixture of expression vectors for target proteins using Lipofectamine 2000 (Thermo Fisher Scientific). The next day, nanoluciferase activity was determined every 2 min up to 1 h at room temperature using the Nano-Glo assay system (Promega) according to the manufacturer’s instructions and FLUOstar Omega (BMG LabTech). Data are expressed as the mean ± S.D. The TGF-β family ligands used in the assay are summarized in Supplementary Table 2.

### Western blot analysis

Whole-cell extracts were prepared using RIPA buffer (Nacalai Tesuque, Kyoto, Japan) containing a protease inhibitor cocktail, separated by electrophoresis in precast 7.5% polyacrylamide gels (Nacalai Tesuque) and transferred to polyvinyl difluoride membranes (Merck, Darmstadt, Germany). The membranes were immediately placed in 5% skim milk (Nacalai Tesuque) in TBS-T buffer (10 mM Tris, 100 mM NaCl, and 0.1% Tween 20) for blocking. The membranes were incubated with a primary antibody at room temperature for 1 h or 4 °C overnight and a secondary antibody conjugated with horseradish peroxidase (Cell Signaling Technology, Danvers, MA, USA) at room temperature for an additional 1 h. Chemiluminescence was detected by the ChemDock XRS+ system (Bio-Rad Laboratories). The antibodies used are listed in table Supplementary Table 3.

### Flow cytometry analysis

Cells were dissociated with enzyme-free cell dissociation buffer (Thermo Fisher Scientific) and resuspended in PBS with 2% FBS at 1 × 10^6^ cells/ml. The cells were stained with a primary antibody at 4 °C for 30 min and a secondary antibody conjugated with Alexa Fluor 647 (Thermo Fisher Scientific) at 4 °C for an additional 30 min. According to the manufacturer’s instructions, the stained cells were analyzed with a BD Accuri C6 flow cytometer (BD Biosciences, San Jose, CA, USA) and C6 software (BD Biosciences) for 5000 events per condition (Supplementary Fig. 8). The antibodies used are listed in Supplementary Table 3.

### BMP-induced heterotopic ossification assay

Human BMP7 (Miltenyi Biotec, Bergisch Gladbach, Germany) and human BMP2 (Corefront Co., Tokyo, Japan) were transplanted into mice as pellets (4 mm diameter) with collagen sponge as a carrier. Collagen pellets were made by hollowing out CollaTape (Zimmer Biomet Dental, Palm Beach Gardens, FL, USA) with a biopsy trepan. An incision was made to expose the quadriceps muscle on the skin and epimysium in male C57BL/6 at 9-week-old (CLEA Japan, Inc, Tokyo, Japan), and a collagen pellet containing human BMP7 or human BMP2 was implanted into the muscle tissue. The mice were subcutaneously injected with rat IgG2a (10 mg/kg, Bio X Cell, Lebanon, NH, USA) or Rm0443 (10 mg/kg) once a week.

### Micro-computed tomography (μCT) analysis

Heterotopic ossification in mice was scanned using CosmoScan GX (Rigaku, Yamanashi, Japan) with a field of view (FOV) of 45 (Fig. 5f) or 60 (Figs. 1d and 5a), X-energy set at 90 kV and 88 μA and an 18-s exposure time. Three-dimensional images were reconstructed from the μCT data obtained using CosmoScan GX software. Quantitative analysis of the area of heterotopic ossification was performed using Analyze 12.0 software (AnalyzeDirect, Inc., Overland Park, KS, USA).

### Histological analysis

The quadriceps muscles containing collagen pellets were fixed with 4% paraformaldehyde-containing phosphate-buffered solution (Nacalai Tesuque) at 4 °C overnight, embedded in paraffin, and used to prepare sections at a thickness of 4 μm using a Leica RM 2125RT rotary microtome (Leica Biosystems, Buffalo Grove, IL, USA). The sections were stained with hematoxylin–eosin and Alcian blue and analyzed with a BZ-9000 microscope (Keyence, Osaka, Japan).

### Protein expression and purification

The gene fragment encoding the extracellular domain of ALK2 (residues 21–123) was synthesized (Thermo Fisher Scientific) and cloned into pET-28b(+) (Merck) for bacterial expression of His-tagged ALK2 (ALK2-His). The gene fragments encoding the variable region of the Rm0443 antibody were synthesized (Thermo Fisher Scientific) and cloned into the expression vector pCMA between the signal sequence and the constant region of human IgG1κ for expression of the rat/human chimeric Rm0443 antibody, which was used in the structural study. ALK2-His was overexpressed in the *Escherichia coli* strain SHuffle T7 (New England Biolabs, Beverly, MA, USA) by induction with 0.1 mM isopropyl β-D-thiogalactopyranoside at 16 °C. The harvested cells were resuspended in lysis buffer (50 mM HEPES (pH 8.0), 500 mM NaCl, 5% glycerol, 20 mM imidazole, Roche cOmplete EDTA-free Protease Inhibitor Cocktail), lysed by sonication, and clarified by centrifugation. The supernatant was then purified using a HisTrap FF crude column (GE Healthcare) and a HiLoad 16/600 Superdex 200 pg column (GE Healthcare). The chimeric Rm0443 antibody was overexpressed in FreeStyle 293F cells (Thermo Fisher Scientific). The culture supernatant was filtered, captured by MabSelect SuRe (GE Healthcare), and then eluted with elution buffer (2 M arginine-HCl, pH 4.0). The eluate was further purified using a Bio-Scale CHT-Type 1 column (Bio-Rad Laboratories). The Fab fragments of the purified antibody were prepared using either a Fab Preparation Kit (Thermo Fisher Scientific) or papain (Merck) and a HiTrap Protein A HP column (GE Healthcare).

### Crystallization and structure determination

Purified ALK2 was mixed with the Rm0443 Fab fragment and concentrated to 2.4 mg/ml. The crystal of the ALK2-Rm0443 complex was obtained with sitting drop vapor diffusion with a precipitant solution containing 2% tacsimate (pH 7.0), 0.1 M HEPES (pH 7.5), and 20% PEG3,350 at 25°C. X-ray diffraction datasets were collected at beamline BL1A at the Photon Factory (Ibaraki, Japan). The datasets were processed and scaled with XDS^56^ and the CCP4 program suite^57^. The crystal of the ALK2-Rm0443 complex belonged to space group C2. The initial phases were obtained with Phaser^58^ using homology models. The ALK2 search model was modeled using Modeller^59^ in Discovery Studio (Dassault Systems) using ALK3 (PDB code 2QJB) as a template, while that of the Rm0443 Fab fragment was modeled using Bioluminate^60^ (Schrödinger). Model refinement was carried out using Refmac^61^ and Coot^62^. All molecular graphics were prepared with PyMOL (The PyMOL Molecular Graphics System, Version 2.0 Schrödinger, LLC.). The model was refined to 2.6 Å resolution with an Rwork = 24.5% and an Rfree = 27.0%. Data collection and refinement statistics are shown in Supplementary Table 4.

### Surface plasmon response analysis

Antibody avidity to the ALK2 protein was measured on a BIAcore T200 (Cytiva, Marlborough, MA, USA). Anti-histidine antibody (Cytiva) was immobilized on a CM5 sensor chip (Cytiva). The His-tagged and Fc-tagged human or mouse ALK2 protein (Sino Biological Inc., 10 μg/ml) was captured. An antibody dilution series (0.195–50 nM) was injected in single-cycle kinetic mode with 300 s of association and 1800 s of dissociation at a 30 μl/min flow rate. The sensors were regenerated by two rounds of 30-s injection of glycine HCl, pH 1.5, at 10 μl /min. The data was analyzed using the bivalent analyte model.

### Immunocytochemistry

The following antibodies were used for immunocytochemistry: anti-V5 antibody (clone V5005, Nacalai Tesuque) and Rm0443. The target proteins were visualized using an Alexa Fluor 488- or 594-conjugated secondary antibody (Thermo Fisher Scientific). Digital images were obtained using a BZ-9000 (Keyence). The antibodies used are listed in Supplementary Table 3.

### Generation of mouse and human ALK2(R206H) FlEx KI mice

Mouse Alk2(R206H) FlEx KI mice and human ALK2(R206H) FlEx KI mice were established using the FlEx system^32,63^. We constructed targeting vectors to express mouse and human ALK2(R206H) cDNA in a Cre-dependent manner, and the constructs contained a 5’ arm and a 3’ arm for coding exon 1 of the *ACVR1* gene (Supplementary Fig. 5a). Two types of FlEx mice in a C57BL/6J background were generated by microinjection with the Cas9 protein, guide RNA and the targeting vector by the Department of Anatomy and Embryology, Faculty of Medicine, University of Tsukuba and Charles River K. K.

### Heterotopic ossification assay in mouse models of FOP

CAG-cre/Esr1 mice expressed the Cre:ERα protein under control of the CAG promoter (The Jackson Laboratory, Bar Harbor, ME, USA: stock #004682)^64^. The mice were crossed with human ALK2(R206H) FlEx KI mice to develop CAG-cre/Esr1;hALK2(R206H) FlEx KI mice. Local DNA recombination was induced by injecting 2.9E + 8 PFU of Cre-expressing adenovirus (SignaGen, Rockville, MD, USA) into the hamstrings of mouse ALK2(R206H) FlEx KI mice. Tamoxifen (Merck) was dissolved at a concentration of 25 mg/ml and intraperitoneally injected at 100 mg/kg/day to induce DNA recombination. Pinch injury of the hamstrings was made using a needle holder (TKZ-HB2216, Takasago Medical Industry Co., Ltd., Tokyo, Japan), and pressure was applied for 10 s three times a day. All mice analyzed were maintained on the C57BL/6 background, fed diet CE-2 (CLEA Japan, Inc), and housed under a SPF condition (12 h light/dark cycle, 50 % humidity, and 23 °C) with free access to food and water.

### Statistics and reproducibility

At least two or three replicates were analyzed in each independent experiment to ensure the experimental data were reliable. Comparisons were performed using unpaired one-way ANOVA and the unpaired *t* test using GraphPad Prism 9 software (GraphPad Software, Inc., San Diego, CA, USA). The IC50 values were calculated using GraphPad Prism 9 software. The results are expressed as the mean ± S.D. Statistical significance is indicated (*P < 0.05, **P < 0.01, ***P < 0.001, and ****P < 0.0001).

### Reporting summary

Further information on research design is available in the Nature Portfolio Reporting Summary linked to this article.

### Supplementary information

Supplementary Information

Reporting Summary

### Source data

Source data

## Data Availability

The coordinates of the refined model and the structure factor have been deposited in the Protein Data Bank (PDB) under the accession code 7YRU. The PDB codes that are referred in this study are available in the Protein Data Bank under accession codes 2GOO, 1IGT, 3Q4U, 2QJB, and 3NH7. All data generated or analyzed during this study are included in this published article (and its Supplementary Information files). Source data are provided with this paper.
