# Publisher Correction: A blocking monoclonal antibody reveals dimerization of intracellular domains of ALK2 associated with genetic disorders

**DOI:** 10.1038/s41467-023-41873-8

**Published:** 2023-09-28

**Authors:** Takenobu Katagiri, Sho Tsukamoto, Mai Kuratani, Shinnosuke Tsuji, Kensuke Nakamura, Satoshi Ohte, Yoshiro Kawaguchi, Kiyosumi Takaishi

**Affiliations:** 1 Division of Biomedical Sciences, Research Center for Genomic Medicine, Saitama Medical University, 1397-1 Yamane, Hidaka-shi, Saitama 350-1241 Japan; 2 Project of Clinical and Basic Research for FOP, Saitama Medical University, 1397-1 Yamane, Hidaka-shi, Saitama 350-1241 Japan; 3 Specialty Medicine Research Laboratories I, R&D Division, Daiichi Sankyo Co., Ltd., 1-2-58 Hiromachi, Shinagawa-ku, Tokyo, 140-8710 Japan; 4 Modality Research Laboratories, Biologics Division, Daiichi Sankyo Co., Ltd., 1-2-58 Hiromachi, Shinagawa-ku, Tokyo, 140-8710 Japan; 5 Present Address: Graduate School of Pharmaceutical Sciences, Kitasato University, 5-9-1 Shirokane, Minato-ku, Tokyo, 108-8641 Japan

**Keywords:** Translational research, Molecular medicine, Growth factor signalling

Correction to: *Nature Communications* 10.1038/s41467-023-38746-5, published online 25 May 2023

The Supplementary Information associated with this Article contained text highlights corresponding to tracked changes that were made during revision. The HTML has been updated to include the correct version of the Supplementary Information, where highlights have been removed.

### Supplementary information

Updated Supplementary Information

Incorrect Supplementary Information

