# Supplementary material for: A blocking monoclonal antibody reveals dimerization of intracellular domains of ALK2 associated with genetic disorders
Source: Nat Commun. 2023 May 25;14:2960. doi: 10.1038/s41467-023-38746-5 (PMC10212922; doi:10.1038/s41467-023-38746-5)
Supplement: Supplementary file 1 — Supplementary Information [file 41467_2023_38746_MOESM1_ESM.pdf]

Supplemental Table 1. Plasmid list.

Plasmid name	cDNA sequence	Vector	Promoter	Epitope tag	Reference
mALK1	Mouse ALK1 WT	pcDEF3	EF1-α	C-terminal V5	Fujimoto et al. 2015
mALK2	Mouse ALK2 WT	pcDEF3	EF1-α	C-terminal V5	This manuscript
mALK3	Mouse ALK3 WT	pcDEF3	EF1-α	C-terminal V5	Fujimoto et al. 2015
mALK4	Mouse ALK4 WT	pcDEF3	EF1-α	C-terminal V5	This manuscript
mALK5	Mouse ALK5 WT	pcDEF3	EF1-α	C-terminal V5	This manuscript
mALK6	Mouse ALK6 WT	pcDEF3	EF1-α	C-terminal V5	Fujimoto et al. 2015
mALK7	Mouse ALK7 WT	pcDEF3	EF1-α	C-terminal V5	This manuscript
hALK1-EGFP	Human ALK1 WT	pcDEF3	EF1-α	C-terminal EGFP	This manuscript
hALK2-EGFP	Human ALK2 WT	pcDEF3	EF1-α	C-terminal EGFP	This manuscript
hALK3-EGFP	Human ALK3 WT	pcDEF3	EF1-α	C-terminal EGFP	This manuscript

hALK4-EGFP	Human ALK4 WT	pcDEF3	EF1-α	C-terminal EGFP	This manuscript
hALK5-EGFP	Human ALK5 WT	pcDEF3	EF1-α	C-terminal EGFP	This manuscript
hALK6-EGFP	Human ALK6 WT	pcDEF3	EF1-α	C-terminal EGFP	This manuscript
hALK7-EGFP	Human ALK7 WT	pcDEF3	EF1-α	C-terminal EGFP	This manuscript
hALK1	Human ALK1 WT	pcDEF3	EF1-α	C-terminal V5	This manuscript
hALK2	Human ALK2 WT	pcDEF3	EF1-α	C-terminal V5	Fukuda et al. 2009
hALK3	Human ALK3 WT	pcDEF3	EF1-α	C-terminal V5	This manuscript
hALK4	Human ALK4 WT	pcDEF3	EF1-α	C-terminal V5	This manuscript
hALK5	Human ALK5 WT	pcDEF3	EF1-α	C-terminal V5	This manuscript
hALK6	Human ALK6 WT	pcDEF3	EF1-α	C-terminal V5	This manuscript
hALK7	Human ALK7 WT	pcDEF3	EF1-α	C-terminal V5	This manuscript
Monkey ALK2	Monkey ALK2 WT	pcDEF3	EF1-α	C-terminal V5	This manuscript
Elephant ALK2	Elephant ALK2 WT	pcDEF3	EF1-α	C-terminal V5	This manuscript
Horse ALK2	Horse ALK2 WT	pcDEF3	EF1-α	C-terminal V5	This manuscript
Dog ALK2	Dog ALK2 WT	pcDEF3	EF1-α	C-terminal V5	This manuscript

Beaver ALK2	Beaver ALK2 WT	pcDEF3	EF1-α	C-terminal V5	This manuscript
Rat ALK2	Rat ALK2 WT	pcDEF3	EF1-α	C-terminal V5	This manuscript
Gerbil ALK2	Gerbil ALK2 WT	pcDEF3	EF1-α	C-terminal V5	This manuscript
Chicken ALK2	Chicken ALK2 WT	pcDEF3	EF1-α	C-terminal V5	This manuscript
Human K25E	Human ALK2 K25E	pcDEF3	EF1-α	C-terminal V5	This manuscript
Human I59V	Human ALK2 I59V	pcDEF3	EF1-α	C-terminal V5	This manuscript
Human H64R	Human ALK2 H64R	pcDEF3	EF1-α	C-terminal V5	This manuscript
Human I103V	Human ALK2 I103V	pcDEF3	EF1-α	C-terminal V5	This manuscript
Human Q106R	Human ALK2 Q106R	pcDEF3	EF1-α	C-terminal V5	This manuscript
Human T113S	Human ALK2 T113S	pcDEF3	EF1-α	C-terminal V5	This manuscript
Rat R64H	Rat ALK2 R64H	pcDEF3	EF1-α	C-terminal V5	This manuscript
Elephant Y63F	Elephant ALK2 Y63F	pcDEF3	EF1-α	C-terminal V5	This manuscript
Chicken A55F/K56H	Chicken ALK2 A55F/K56H	pcDEF3	EF1-α	C-terminal V5	This manuscript
L196P	Human ALK2 L196P	pcDEF3	EF1-α	C-terminal V5	Ohte et al. 2011
PF197_8L	Human ALK2 PF197_8L	pcDEF3	EF1-α	C-terminal V5	Fujimoto et al. 2015

R202I	Human ALK2 R202I	pcDEF3	EF1-α	C-terminal V5	Fujimoto et al. 2015
R206H	Human ALK2 R206H	pcDEF3	EF1-α	C-terminal V5	Fukuda et al. 2009
Q207E	Human ALK2 Q207E	pcDEF3	EF1-α	C-terminal V5	Fujimoto et al. 2015
R258G	Human ALK2 R258G	pcDEF3	EF1-α	C-terminal V5	Machiya et al. 2018
R258S	Human ALK2 R258S	pcDEF3	EF1-α	C-terminal V5	Fujimoto et al. 2015
G325A	Human ALK2 G325A	pcDEF3	EF1-α	C-terminal V5	Fujimoto et al. 2015
G328E	Human ALK2 G328E	pcDEF3	EF1-α	C-terminal V5	Fujimoto et al. 2015
G328R	Human ALK2 G328R	pcDEF3	EF1-α	C-terminal V5	Fujimoto et al. 2015
G328W	human ALK2 G328W	pcDEF3	EF1-α	C-terminal V5	Fujimoto et al. 2015
G356D	Human ALK2 G356D	pcDEF3	EF1-α	C-terminal V5	Fukuda et al. 2008
R375P	Human ALK2 R375P	pcDEF3	EF1-α	C-terminal V5	Fujimoto et al. 2015
WT-LgBiT	Human ALK2 WT	N196 pBiT1.1-C [TK/LgBiT]	HSV-TK	C-terminal LgBiT	This manuscript
WT-SmBiT	Human ALK2 WT	N197 pBiT2.1-C [TK/SmBiT]	HSV-TK	C-terminal SmBiT	This manuscript
R206H-LgBiT	Human ALK2 R206H	N196 pBiT1.1-C [TK/LgBiT]	HSV-TK	C-terminal LgBiT	This manuscript
R206H-SmBiT	Human ALK2 R206H	N197 pBiT2.1-C [TK/SmBiT]	HSV-TK	C-terminal SmBiT	This manuscript
ActR-IIB	Mouse ActR-IIB WT	N197 pBiT2.1-C [TK/SmBiT]	HSV-TK	C-terminal FLAG	This manuscript

Q207E-Lg-BiT	Human ALK2 Q207E	N196 pBiT1.1-C [TK/LgBiT]	HSV-TK	C-terminal LgBiT	This manuscript
Q207E-SmBiT	Human ALK2 R258G	N197 pBiT2.1-C [TK/SmBiT]	HSV-TK	C-terminal SmBiT	This manuscript
R258G-LgBiT	Human ALK2 R258G	N196 pBiT1.1-C [TK/LgBiT]	HSV-TK	C-terminal LgBiT	This manuscript
R258G-SmBiT	Human ALK2 R258G	N197 pBiT2.1-C [TK/SmBiT]	HSV-TK	C-terminal SmBiT	This manuscript
R258S-LgBiT	Human ALK2 R258S	N196 pBiT1.1-C [TK/LgBiT]	HSV-TK	C-terminal LgBiT	This manuscript
R258S-SmBiT	Human ALK2 R258S	N197 pBiT2.1-C [TK/SmBiT]	HSV-TK	C-terminal SmBiT	This manuscript
G325A-LgBiT	Human ALK2 G325A	N196 pBiT1.1-C [TK/LgBiT]	HSV-TK	C-terminal LgBiT	This manuscript
G325A-SmBiT	Human ALK2 G325A	N197 pBiT2.1-C [TK/SmBiT]	HSV-TK	C-terminal SmBiT	This manuscript
G328E-LgBiT	Human ALK2 G328E	N196 pBiT1.1-C [TK/LgBiT]	HSV-TK	C-terminal LgBiT	This manuscript
G328E-SmBiT	Human ALK2 G328E	N197 pBiT2.1-C [TK/SmBiT]	HSV-TK	C-terminal SmBiT	This manuscript
G356D-LgBiT	Human ALK2 G356D	N196 pBiT1.1-C [TK/LgBiT]	HSV-TK	C-terminal LgBiT	This manuscript
G356D-SmBiT	Human ALK2 G356D	N197 pBiT2.1-C [TK/SmBiT]	HSV-TK	C-terminal SmBiT	This manuscript
G328V-LgBiT	Human ALK2 G328V	N196 pBiT1.1-C [TK/LgBiT]	HSV-TK	C-terminal LgBiT	This manuscript
G328V-SmBiT	Human ALK2 G328V	N197 pBiT2.1-C [TK/SmBiT]	HSV-TK	C-terminal SmBiT	This manuscript
K400E-LgBiT	Human ALK2 K400E	N196 pBiT1.1-C [TK/LgBiT]	HSV-TK	C-terminal LgBiT	This manuscript
K400E-SmBiT	Human ALK2 K400E	N197 pBiT2.1-C [TK/SmBiT]	HSV-TK	C-terminal SmBiT	This manuscript

Q207D-LgBiT	Human ALK2 Q207D	N196 pBiT1.1-C [TK/LgBiT]	HSV-TK	C-terminal LgBiT	This manuscript
Q207D-SmBiT	Human ALK2 Q207D	N197 pBiT2.1-C [TK/SmBiT]	HSV-TK	C-terminal SmBiT	This manuscript
WT/H64R-SmBiT	Human ALK2 H64R	N197 pBiT2.1-C [TK/SmBiT]	HSV-TK	C-terminal SmBiT	This manuscript
R206H/H64R-SmBiT	Human ALK2 H64R-R206H	N197 pBiT2.1-C [TK/SmBiT]	HSV-TK	C-terminal SmBiT	This manuscript
WT-LgBiT	Human ALK2 WT	pcDEF3	EF1-α	C-terminal LgBiT	This manuscript
WT-HiBiT	Human ALK2 WT	pcDEF3	EF1-α	C-terminal HiBiT	This manuscript
R206H-LgBiT	Human ALK2 R206H	pcDEF3	EF1-α	C-terminal LgBiT	This manuscript
R206H-HiBiT	Human ALK2 R206H	pcDEF3	EF1-α	C-terminal HiBiT	This manuscript
ActR-IIA	Mouse ActR-IIA WT	N197 pBiT2.1-C [TK/SmBiT]	HSV-TK	C-terminal FLAG	This manuscript
BMPR-II (long)	Human BMPR-II (long) WT	N197 pBiT2.1-C [TK/SmBiT]	HSV-TK	C-terminal FLAG	This manuscript
BMPR-II (short)	Human BMPR-II (short) WT	N197 pBiT2.1-C [TK/SmBiT]	HSV-TK	C-terminal FLAG	This manuscript
AMHR-II	Mouse AMHR-II WT	N197 pBiT2.1-C [TK/SmBiT]	HSV-TK	C-terminal FLAG	This manuscript
TβR-II	Mouse TβR-II WT	N197 pBiT2.1-C [TK/SmBiT]	HSV-TK	C-terminal FLAG	This manuscript
ActR-IIB(KR)	Mouse ActR-IIB KR	N197 pBiT2.1-C [TK/SmBiT]	HSV-TK	C-terminal FLAG	This manuscript
ALK2 WT	Human ALK2 WT	N197 pBiT2.1-C [TK/SmBiT]	HSV-TK	C-terminal V5	This manuscript
ALK2 R206H	Human ALK2 R206H	N197 pBiT2.1-C [TK/SmBiT]	HSV-TK	C-terminal V5	This manuscript

ActR-IIB-LgBiT	Mouse ActR-IIB WT	N196 pBiT1.1-C [TK/LgBiT]	HSV-TK	C-terminal LgBiT	This manuscript
ActR-IIB-SmBiT	Mouse ActR-IIB WT	N197 pBiT2.1-C [TK/SmBiT]	HSV-TK	C-terminal SmBiT	This manuscript
WT(KR)-LgBiT	Human ALK2 K235R	N196 pBiT1.1-C [TK/LgBiT]	HSV-TK	C-terminal LgBiT	This manuscript
WT(KR)-SmBiT	Human ALK2 K235R	N197 pBiT2.1-C [TK/SmBiT]	HSV-TK	C-terminal SmBiT	This manuscript
RH(KR)-LgBiT	Human ALK2 R206H/K235R	N196 pBiT1.1-C [TK/LgBiT]	HSV-TK	C-terminal LgBiT	This manuscript
RH(KR)-SmBiT	Human ALK2 R206H/K235R	N197 pBiT2.1-C [TK/SmBiT]	HSV-TK	C-terminal SmBiT	This manuscript
WT(KR)	Human ALK2 K235R	N197 pBiT2.1-C [TK/SmBiT]	HSV-TK	C-terminal V5	This manuscript
Q207E	Human ALK2 Q207E	N197 pBiT2.1-C [TK/SmBiT]	HSV-TK	C-terminal V5	This manuscript
Q207D	Human ALK2 Q207D	N197 pBiT2.1-C [TK/SmBiT]	HSV-TK	C-terminal V5	This manuscript
G328V	Human ALK2 G328V	N197 pBiT2.1-C [TK/SmBiT]	HSV-TK	C-terminal V5	This manuscript
K400E	Human ALK2 K400E	N197 pBiT2.1-C [TK/SmBiT]	HSV-TK	C-terminal V5	This manuscript
G325A	Human ALK2 G325A	N197 pBiT2.1-C [TK/SmBiT]	HSV-TK	C-terminal V5	This manuscript
hALK2(ΔICD)-LgBiT	Human ALK2 F149_C509del	N196 pBiT1.1-C [TK/LgBiT]	HSV-TK	C-terminal LgBiT	This manuscript
hALK2(ΔICD)-SmBiT	Human ALK2 F149_C509del	N197 pBiT2.1-C [TK/SmBiT]	HSV-TK	C-terminal SmBiT	This manuscript
ActR-IIB(ΔICD)-FLAG	Mouse ActR-IIB P165_I536del	N197 pBiT2.1-C [TK/SmBiT]	HSV-TK	C-terminal FLAG	This manuscript

mR206H	Mouse ALK2 R206H	pcDEF3	EF1-α	C-terminal V5	This manuscript
Chimera WT	Human ALK2 ECD + mouse ALK2 ICD	pcDEF3	EF1-α	C-terminal V5	This manuscript
Chimera R206H	Human ALK2 ECD + mouse ALK2 R206H ICD	pcDEF3	EF1-α	C-terminal V5	This manuscript
hR206H/D182E	Human ALK2 R206H/D182E	pcDEF3	EF1-α	C-terminal V5	This manuscript
hR206H/P330S	Human ALK2 R206H/P330S	pcDEF3	EF1-α	C-terminal V5	This manuscript
WT-P330S-LgBiT	Human ALK2 P330S	N196 pBiT1.1-C [TK/LgBiT]	HSV-TK	C-terminal LgBiT	This manuscript
WT-P330S-SmBiT	Human ALK2 P300S	N197 pBiT2.1-C [TK/SmBiT]	HSV-TK	C-terminal SmBiT	This manuscript
R206H-P330S-LgBiT	Human ALK2 R206H-P330S	N196 pBiT1.1-C [TK/LgBiT]	HSV-TK	C-terminal LgBiT	This manuscript
R206H-P330S-SmBiT	Human ALK2 R206H-P300S	N197 pBiT2.1-C [TK/SmBiT]	HSV-TK	C-terminal SmBiT	This manuscript
WT-P330S	Human ALK2 WT-P330S	pcDEF3	EF1-α	C-terminal V5	This manuscript
R206H-P330C	Human ALK2 R206H-P330C	pcDEF3	EF1-α	C-terminal V5	This manuscript
R206H-P330F	Human ALK2 R206H-P330F	pcDEF3	EF1-α	C-terminal V5	This manuscript
R206H-P330I	Human ALK2 R206H-P330I	pcDEF3	EF1-α	C-terminal V5	This manuscript

R206H-P330V	Human ALK2 R206H-P330V	pcDEF3	EF1-α	C-terminal V5	This manuscript
R206H-P330K	Human ALK2 R206H-P330K	pcDEF3	EF1-α	C-terminal V5	This manuscript
R206H-P330L	Human ALK2 R206H-P330L	pcDEF3	EF1-α	C-terminal V5	This manuscript
R206H-P330Q	Human ALK2 R206H-P330Q	pcDEF3	EF1-α	C-terminal V5	This manuscript
R206H-P330T	Human ALK2 R206H-P330T	pcDEF3	EF1-α	C-terminal V5	This manuscript
R206H-P330Y	Human ALK2 R206H-P330Y	pcDEF3	EF1-α	C-terminal V5	This manuscript
R206H-P330A	Human ALK2 R206H-P330A	pcDEF3	EF1-α	C-terminal V5	This manuscript
R206H-P330D	Human ALK2 R206H-P330D	pcDEF3	EF1-α	C-terminal V5	This manuscript
R206H-P330E	Human ALK2 R206H-P330E	pcDEF3	EF1-α	C-terminal V5	This manuscript
R206H-P330G	Human ALK2 R206H-P330G	pcDEF3	EF1-α	C-terminal V5	This manuscript
R206H-P330H	Human ALK2 R206H-P330H	pcDEF3	EF1-α	C-terminal V5	This manuscript
R206H-P330N	Human ALK2 R206H-P330N	pcDEF3	EF1-α	C-terminal V5	This manuscript
R206H-P330R	Human ALK2 R206H-P330R	pcDEF3	EF1-α	C-terminal V5	This manuscript
R206H-P330W	Human ALK2 R206H-P330W	pcDEF3	EF1-α	C-terminal V5	This manuscript
mR206H-S330P	Mouse ALK2 R206H-S330P	pcDEF3	EF1-α	C-terminal V5	This manuscript
ALK2-ECD	Human ALK2 (residues 21–123)				

		pET-28b(+)	T7	C-terminal His	This manuscript
Rm0443_HC	Rm0443 heavy chain	pCMA	CMV	-	This manuscript
Rm0443_LC	Rm0443 light chain	pCMA	CMV	-	This manuscript
Id1 WT4F-luc	Human Id1 BRE	pGL4.26	-	-	Katagiri et al. 2002
					Ohte et al. 2011
					Promega #E8441
phRL-SV40	Renilla	phRL	-	-	Promega #E2231

Supplemental Table 2. Ligand list.

Protein name	Species	Source	Purchase	Catalog#
BMP2	Human	*E. coli*	CoreFront	# CK-B2
BMP7	Human	CHO cell	Miltenyi Biotec	# 130-108-988
BMP9 (GDF2)	Human	CHO cell	PeproTech	# 120-07
GDF5	Mouse	*E. coli*	PeproTech	# 315-24
Activin A	Human/Mouse/Rat	*E. coli*	PeproTech	# 120-14E
Myostatin	Human/Mouse/Rat	*E. coli*	PeproTech	# 120-00

Supplemental Table 3-1. List of antibodies used for immunocytochemistry.

Antibody name	Host species	Clonality	Dilution	Purchase	Catalog#
Anti-ALK2 (Rm0443)	Rat	Monoclonal	10 µg/ml	-	-
Anti-V5	Mouse	Monoclonal (clone V5005)	1:1000	Nacalai Tesque	#04434-94
Anti-rat IgG, Alexa 488	Goat	Polyclonal	1:1000	Thermo Fisher Scientific	#A11006
Anti-mouse IgG, Alexa 594	Goat	Polyclonal	1:1000	Thermo Fisher Scientific	#A11032

Supplemental Table 3-2. List of antibodies used for flow cytometry.

Antibody name	Host species	Clonality	Dilution	Purchase	Catalog#
Anti-ALK2 (Rm0443)	Rat	Monoclonal	10 μg/ml	-	-
IgG2a isotype control	Rat	Monoclonal (clone 54447)	1:1000	R&D	#MAB006
Anti-rat IgG, Alexa 647	Goat	Polyclonal	1:1000	Thermo Fisher Scientific	#A21247

Supplemental Table 3-3. List of antibodies used for western blot.

Antibody name	Host species	Clonality	Dilution	Purchase	Catalog#
Anti-ALK2 (Rm0443)	Rat	Monoclonal	1 μg/ml	-	-
Anti-V5	Rabbit	Monoclonal (clone D3H8Q)	1:1000	Cell Signaling Technology	#13202
Anti- α -Tubulin	Rabbit	Polyclonal	1:1000	Cell Signaling Technology	#2144
Anti-rat IgG, HRP	Goat	Polyclonal	1:1000	Cell Signaling Technology	#7077
Anti-rabbit IgG, HRP	Goat	Polyclonal	1:1000	Cell Signaling Technology	#7074

Supplementary Table 4. X-ray crystallographic data collection and refinement statistics.

	7YRU*
**Data collection**	
Space group	C121
Cell dimensions	
a, *b*, *c* (Å)	119.05, 37.30, 118.64
α, β, γ (°)	90.00, 92.80, 90.00
Resolution (Å)	43.30 - 2.60 (2.72 - 2.60) **
R_{merge}	0.082 (0.384)
$I / \sigma I$	6.8 (1.1)
Completeness (%)	98.30 (99.70)
Redundancy	2.80 (2.90)
**Refinement**	
Resolution (Å)	43.30 - 2.60 (2.67 – 2.60)
No. reflections	15215 (1128)
R_{work} / R_{free}	0.245 (0.270)
No. atoms	
Protein	3858
Ligand/ion	0
Water	55
B-factors	
Protein	49.64
Ligand/ion	
Water	39.76
R.m.s. deviations	
Bond lengths (Å)	0.007
Bond angles (°)	1.245

*Single crystal was used.
**Values in parentheses are for highest-resolution shell.

Suppl. Fig. 1

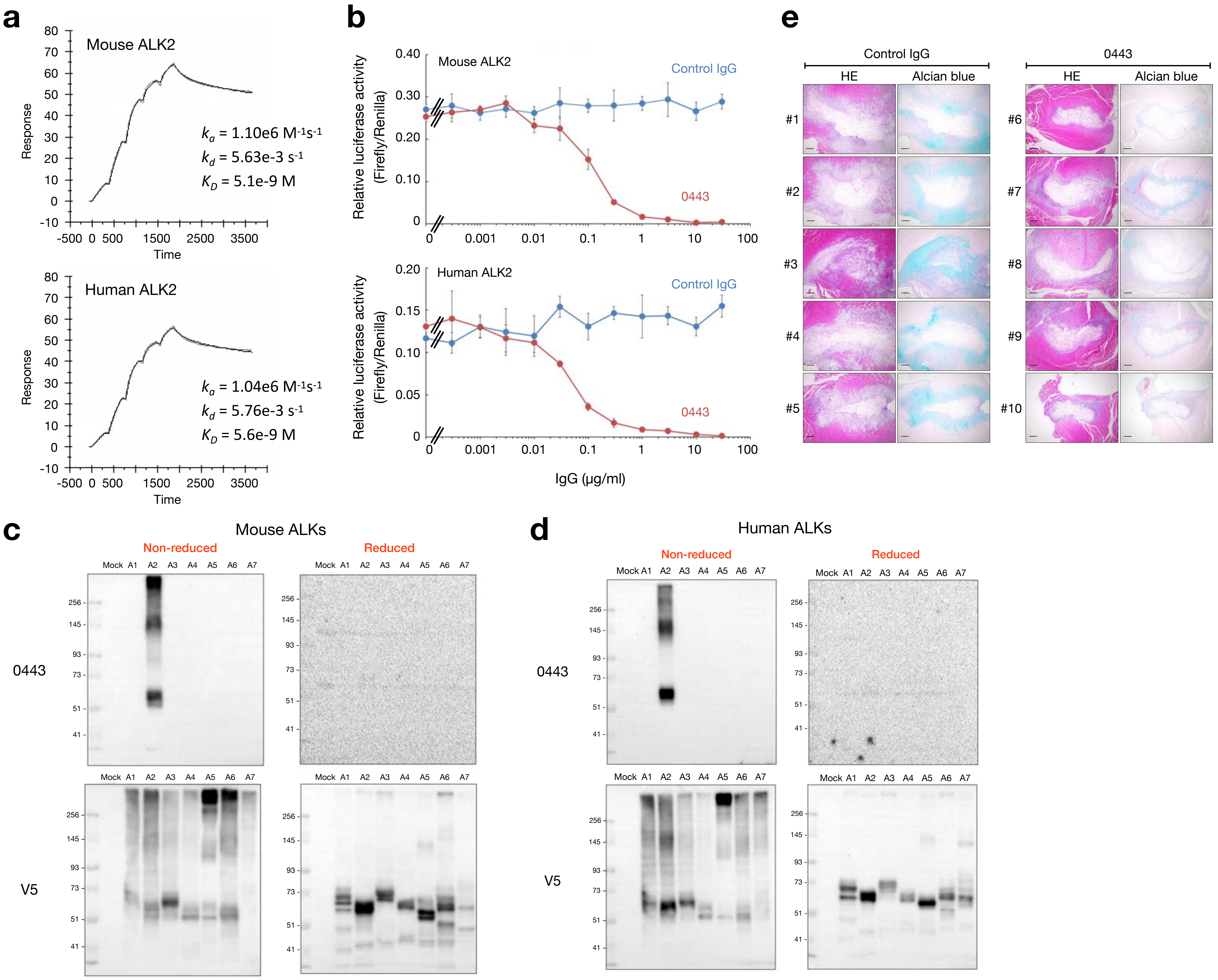

a
Mouse ALK2
Response
Time
k_a = 1.10e6 M-1s-1
k_d = 5.63e-3 s-1
K_D = 5.1e-9 M
Human ALK2
k_a = 1.04e6 M-1s-1
k_d = 5.76e-3 s-1
K_D = 5.6e-9 M
b
Mouse ALK2
Human ALK2
Relative luciferase activity (Firefly/Renilla)
Control IgG
0443
IgG (μg/ml)
e
Control IgG
0443
HE
Alcian blue
#1
#2
#3
#4
#5
#6
#7
#8
#9
#10
c
Mouse ALKs
d
Human ALKs
Non-reduced
Reduced
Mock A1 A2 A3 A4 A5 A6 A7
0443
V5

Supplementary Figure 1. Characterization of Rm0443

a. BIAcore assay with Rm0443 on mouse and human ALK2 ECDs. The binding affinities of Rm0443 for mouse (upper panel) and human (lower panel) ALK2 ECDs were analysed by the BIAcore assay.

b. Dose-dependent inhibition of BMP signaling via mouse and human ALK2 by Rm0443. HEK293A cells were transfected with mouse (upper panel) or human (lower panel) ALK2 and reporter plasmids. The cells were incubated overnight with 10 ng/ml BMP7 and increasing concentrations of control IgG or Rm0443. IC50 values of Rm0443 for mouse and human ALK2 were 0.113 and 0.044 μg/ml, respectively. The lowest concentration on the dose response curve represents the absence of antibodies (n = 3, biologically independent samples). Data were expressed as the mean ± S. D. Source data are provided as a Source Data file.

c and d. Western blot analysis of mouse (c) and human (d) ALK1 through ALK7 under reducing and non-reducing conditions using Rm0443. HEK293A cells were transfected with one of the V5-tagged mouse (c) and human (d) ALK1 through ALK7 constructs. The cell extracts were separated by SDS-PAGE under non-reducing (left panels) and reducing (right panels) conditions and analysed by western blotting using Rm0443 (upper panels) and an antibody against V5-tag (lower panels). Two independent experiments were performed. Source data are provided as a Source Data file.

e. Histological analysis of collagen pellets containing BMP7 in mice. Collagen pellets containing 5 μg of BMP7 were transplanted into the skeletal muscle of wild-type mice, which were injected with 10 mg/kg control IgG or Rm0443 once. On day 7 after

transplantation, the pellets were analysed by histological staining with haematoxylin and eosin (HE) and Alcian blue (n = 5 for control IgG (#1 to #5); n = 5 for Rm0443 (#6 to #10)). The scale bars represent 300 µm.

Suppl. Fig. 2

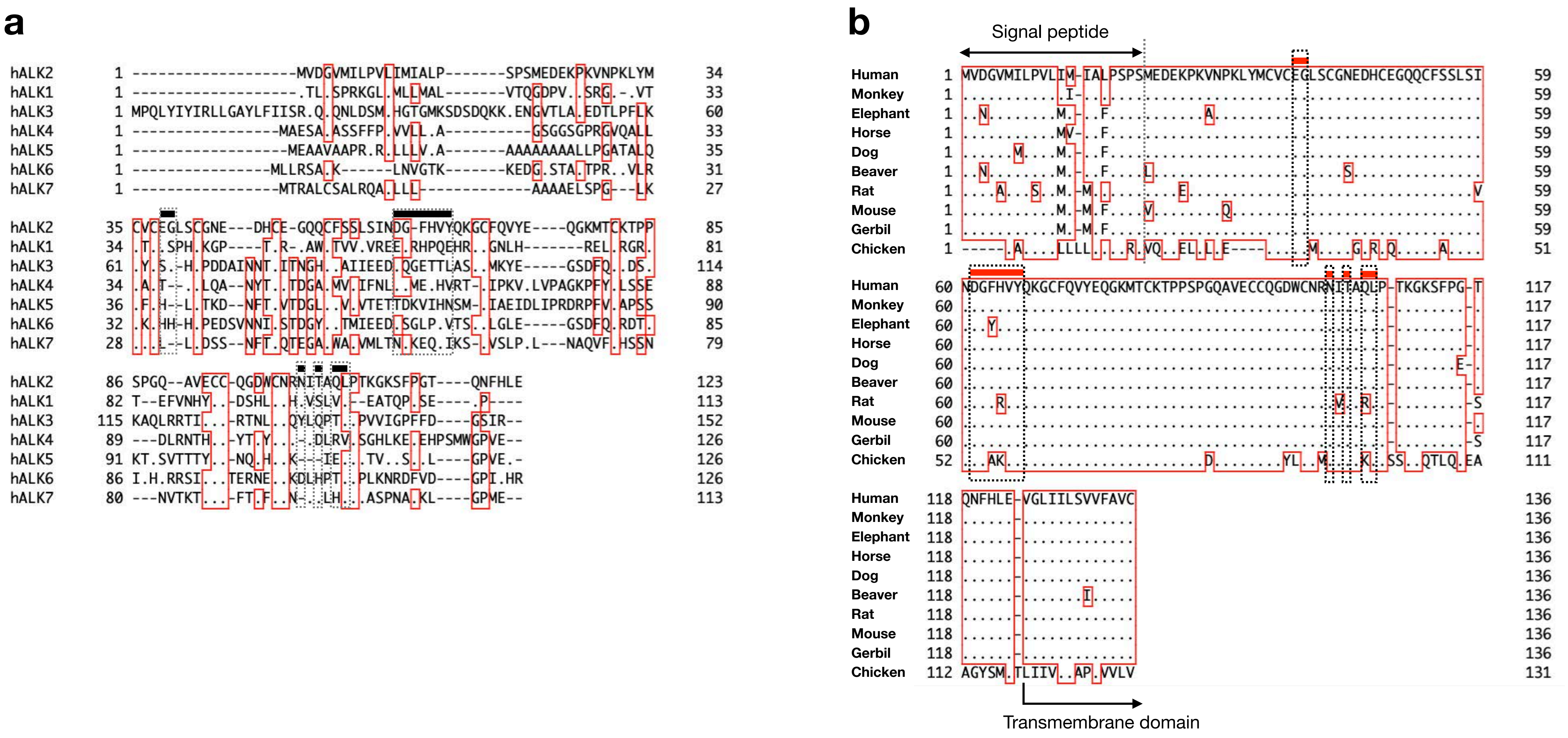
a
hALK2
hALK1
hALK3
hALK4
hALK5
hALK6
hALK7
b
Signal peptide
Human
Monkey
Elephant
Horse
Dog
Beaver
Rat
Mouse
Gerbil
Chicken
Transmembrane domain

Supplementary Figure 2. Alignments of sequences of ALK2 and other ALK receptors.

a. Alignment of the amino acid sequences of human ALK1 to ALK7 ECDs. Amino acid sequences were analyzed using Genetyx Mac ver. 20. Conserved residues among the receptors are boxed in red, and epitopes identified in ALK2 are shown by lines in black.

b. Alignment of the amino acid sequences of ten types of ALK2 ECDs. The amino acid sequences of human (*Homo sapiens*), monkey (*Macaca mulatta*), elephant (*Loxodonta africana*), horse (*Equus caballus*), dog (*Canis lupus familiaris*), beaver (*Castor canadensis*), rat (*Rattus rattus*), mouse (*Mus musculus*), gerbil (*Meriones unguiculatus*), and chicken (*Gallus gallus*) ALK2 proteins were analyzed using Genetyx Mac ver. 20. Epitopes identified by crystallographic analysis are boxed with dotted lines.

Suppl. Fig. 3

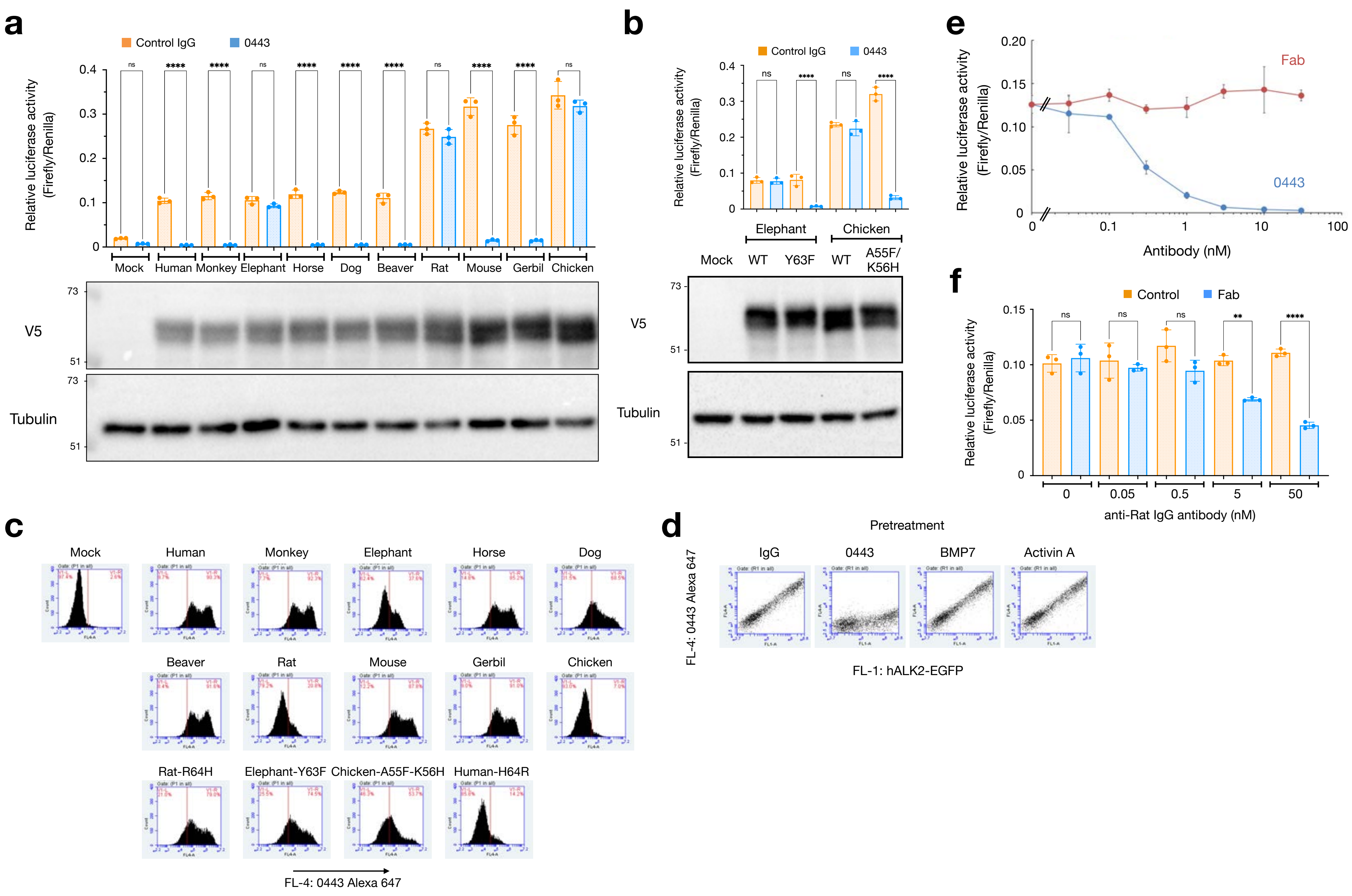
a
Control IgG
0443
Relative luciferase activity (Firefly/Renilla)
Mock
Human
Monkey
Elephant
Horse
Dog
Beaver
Rat
Mouse
Gerbil
Chicken
V5
Tubulin
b
Elephant
Chicken
Mock
WT
Y63F
A55F/K56H
e
Fab
0443
Antibody (nM)
f
Control
anti-Rat IgG antibody (nM)
c
Rat-R64H
Elephant-Y63F
Chicken-A55F-K56H
Human-H64R
FL-4: 0443 Alexa 647
d
Pretreatment
IgG
BMP7
Activin A
FL-1: hALK2-EGFP

Supplementary Figure 3. Analysis of critical residues in ALK2 for binding and inhibition by Rm0443.

a. V5-tagged ALK2 expression vectors were constructed for human, monkey, elephant, horse, dog, beaver, rat, mouse, gerbil, and chicken ALK2 sequences. Each vector was co-transfected with reporter plasmids in HEK293A cells, which were then cultured overnight with 10 ng/ml BMP7 and 10 μg/ml control IgG or Rm0443 (n = 3, biologically independent samples). Data were expressed as the mean ± S. D. Expression levels of ALK2 were determined by western blotting using an antibody against V5-tag. Source data are provided as a Source Data file.

b. Effects of Rm0443 on BMP signaling via elephant and chicken ALK2. Elephant and chicken ALK2 were subjected to the Y63F and A55F/K56H mutations, respectively. HEK293A cells were transfected with elephant and chicken ALK2 expression vectors and reporter plasmids. The cells were treated overnight with 10 ng/ml BMP7 and 3 μg/ml control IgG or Rm0443 (n = 3, biologically independent samples). Data were expressed as the mean ± S. D. ALK2 expression levels were determined by western blotting using an antibody against V5-tag. Source data are provided as a Source Data file.

c. Flow cytometry analysis of the capacity of Rm0443 to bind ALK2. HEK293A cells transfected with one of the ALK2 constructs as indicated in the figure were analysed by flow cytometry analysis using Rm0443 and Alexa Fluor 647-conjugated anti-rat antibody.

d. Flow cytometry analysis of the capacity of Rm0443 to binding ALK2 in cells pre-treated with ligand. HEK293A cells were transfected with human ALK2-EGFP. The cell suspensions were incubated with 1 μg/ml BMP7, 1 μg/ml activin A, and 10 μg/ml

Rm0443 for 30 min. Then, they were incubated with 2 μg/ml Alexa Fluor 647-conjugated Rm0443 for an additional 30 min.

e. The Rm0443 Fab fragment did not inhibit signaling. HEK293A cells were transfected with wild-type human ALK2 with reporter plasmids. The cells were treated with 10 ng/ml BMP7 with increasing concentrations of Rm0443 or the Rm0443 Fab fragment. The lowest concentration on the dose response curve represents the absence of antibodies (n = 3, biologically independent samples). Data were expressed as the mean ± S. D. Source data are provided as a Source Data file.

f. The Rm0443 Fab fragment inhibited signaling in the presence of a secondary antibody. HEK293A cells were transfected with human ALK2(WT) and reporter plasmids. The cells were treated with 10 ng/ml BMP7 and increasing concentrations of a secondary anti-rat antibody in the absence (orange columns) or presence (blue columns) of 200 nM Rm0443 Fab fragment (n = 3, biologically independent samples). Data were expressed as the mean ± S. D. Source data are provided as a Source Data file.

P values are calculated using unpaired one-way ANOVA and indicated significant if $^{**}P < 0.01$ or $^{****}P < 0.0001$.

Suppl. Fig. 4

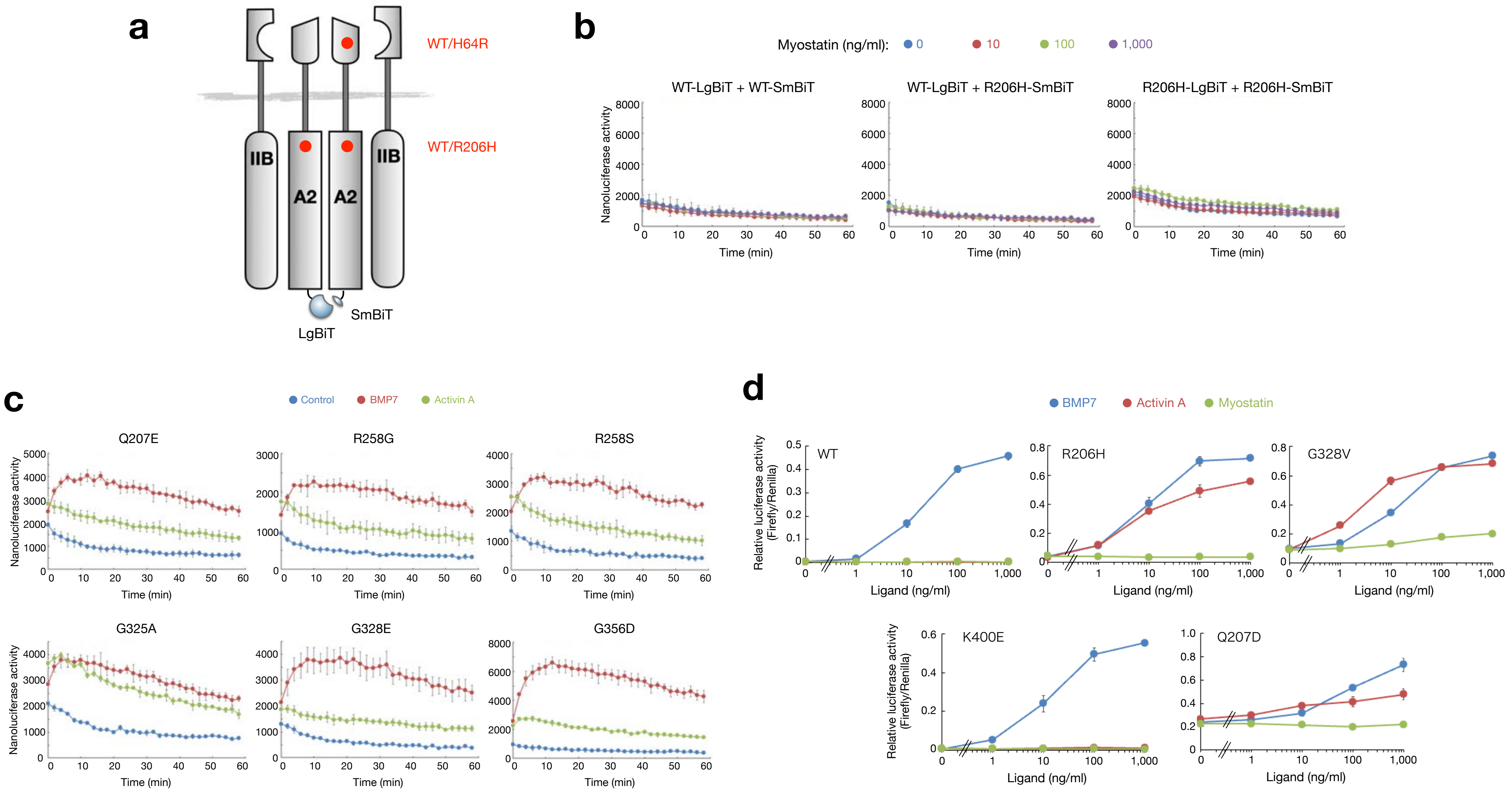
a
WT/H64R
WT/R206H
IIB
A2
A2
IIB
SmBiT
LgBiT
b
Myostatin (ng/ml): 0 10 100 1,000
WT-LgBiT + WT-SmBiT
WT-LgBiT + R206H-SmBiT
R206H-LgBiT + R206H-SmBiT
Nanoluciferase activity
Time (min)
c
Control BMP7 Activin A
Q207E
R258G
R258S
G325A
G328E
G356D
Nanoluciferase activity
Time (min)
d
BMP7 Activin A Myostatin
WT
R206H
G328V
K400E
Q207D
Relative luciferase activity (Firefly/Renilla)
Ligand (ng/ml)

Supplementary Figure 4. NanoBiT assay for monitoring the dimer formation of ALK2 ICDs.

a. Dimer formation of ALK2 ICDs was monitored using NanoBiT, in which nanoluciferase was separated into inactive large (LgBiT) and small (SmBiT) subunits. Each subunit was fused to the C-terminus of wild-type or R206H ALK2. The restored nanoluciferase activity was determined in the presence or absence of FLAG (unrelated)-tagged ActR-IIB. Red dots indicate the H64R and R206H substitutions in ALK2 ECD and ICD, respectively.

b. Dimer formation of ALK2 ICDs in response to myostatin. HEK293A cells were transfected with ALK2-LgBiT and ALK2-SmBiT consisting of wild-type and R206H ALK2 with FLAG (unrelated)-tagged ActR-IIB as indicated and stimulated with 0 (blue), 10 (red), 100 (green), and 1,000 ng/ml (purple) myostatin. (n = 3, biologically independent samples). Data were expressed as the mean ± S. D. Source data are provided as a Source Data file.

c. Dimer formation of ALK2 ICDs associated with disorders in response to ligands. HEK293A cells were transfected with ALK2-LgBiT and ALK2-SmBiT containing Q207E, R258G, R258S, G325A, G328E, and G356D ALK2 with FLAG (unrelated)-tagged ActR-IIB. The cells were stimulated without (blue) or with 100 ng/ml BMP7 (red) or 100 ng/ml activin A (green) (n = 3, biologically independent samples). Data were expressed as the mean ± S. D. Source data are provided as a Source Data file.

d. BMP activity induced by ALK2 in response to ligand stimulation. HEK293A cells were transfected with wild-type ALK2, and R206H, G328V, K400E, and Q207D mutant

ALK2 with reporter plasmids. To avoid saturation of the luciferase activity, the G328V and Q207D ALK2 mutants were diluted 1:5 and 1:100 with the mock plasmid. Cells were stimulated without and with 1, 10, 100, and 1,000 ng/ml BMP7 (blue), activin A (red), and myostatin (green) ($n = 3$, biologically independent samples). Data were expressed as the mean ± S. D. Source data are provided as a Source Data file.

Suppl. Fig. 5

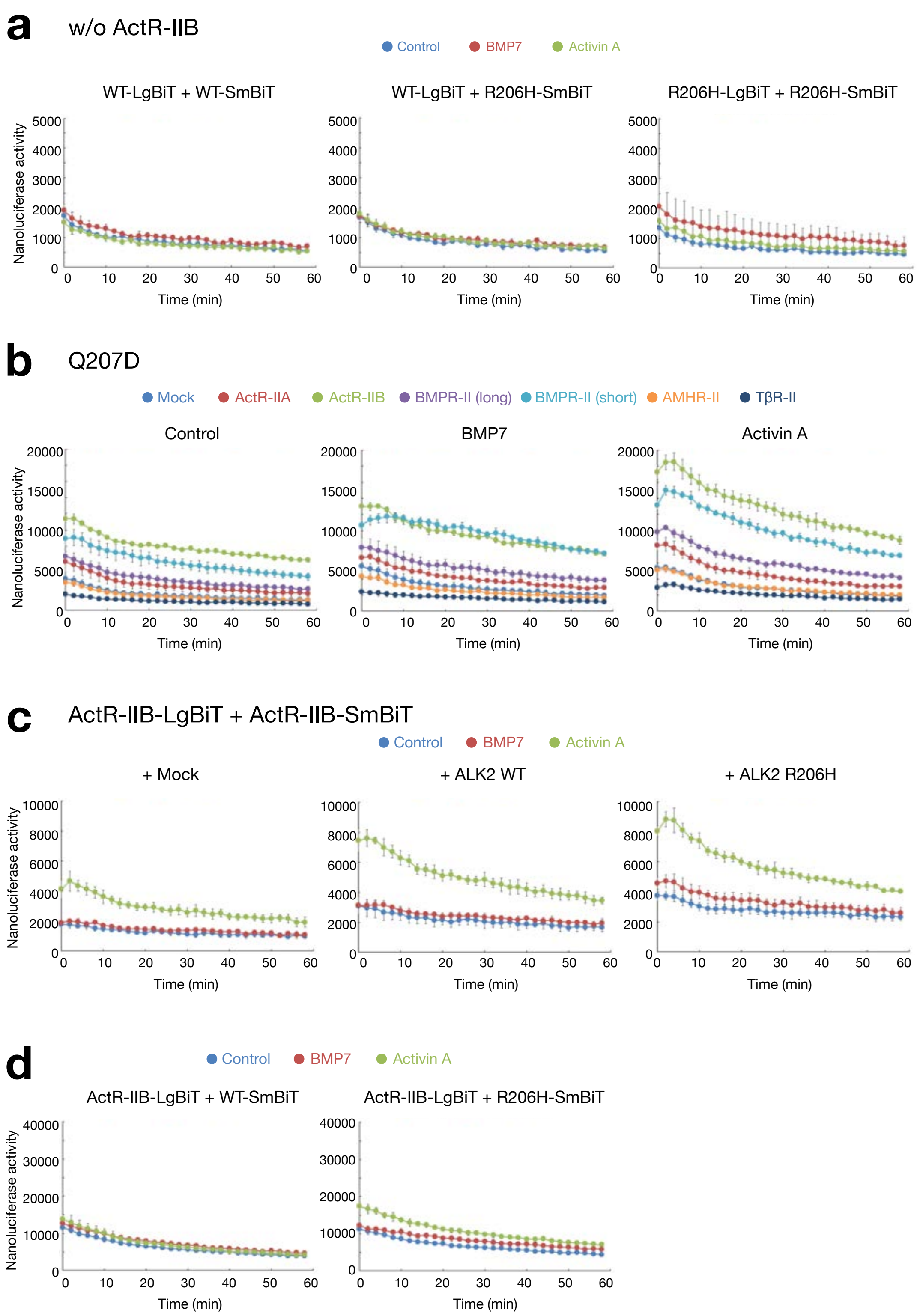
a
w/o ActR-IIB
Control
BMP7
Activin A
WT-LgBiT + WT-SmBiT
WT-LgBiT + R206H-SmBiT
R206H-LgBiT + R206H-SmBiT
Nanoluciferase activity
Time (min)
b
Q207D
Mock
ActR-IIA
ActR-IIB
BMPR-II (long)
BMPR-II (short)
AMHR-II
TβR-II
Control
BMP7
Activin A
Nanoluciferase activity
Time (min)
c
ActR-IIB-LgBiT + ActR-IIB-SmBiT
Control
BMP7
Activin A
+ Mock
+ ALK2 WT
+ ALK2 R206H
Nanoluciferase activity
Time (min)
d
Control
BMP7
Activin A
ActR-IIB-LgBiT + WT-SmBiT
ActR-IIB-LgBiT + R206H-SmBiT
Nanoluciferase activity
Time (min)

Supplementary Figure 5. Type II receptors are required for the dimer formation of ALK2 ICDs.

a. Dimer formation of ALK2 ICDs in the absence of ActR-IIB. HEK293A cells were transfected with ALK2-LgBiT and ALK2-SmBiT containing wild-type and R206H ALK2 without ActR-IIB as indicated and stimulated without (blue) or with 100 ng/ml BMP7 (red) or 100 ng/ml activin A (green) (n = 3, biologically independent samples). Data were expressed as the mean ± S. D. Source data are provided as a Source Data file.

b. Dimer formation of Q207D ALK2 ICDs. HEK293A cells were transfected with ALK2 Q207D-LgBiT and ALK2 Q207D-SmBiT with FLAG (unrelated)-tagged type II receptor. The cells were stimulated without (left) or with 100 ng/ml BMP7 (middle) or 100 ng/ml activin A (right). (n = 3, biologically independent samples). Data were expressed as the mean ± S. D. Source data are provided as a Source Data file.

c. Dimer formation of ActR-IIB ICDs. HEK293A cells were transfected with ActR-IIB-LgBiT and ActR-IIB-SmBiT with mock vector, ALK2 WT-V5, and ALK2 R206H-V5 as indicated and stimulated without (blue) or with 100 ng/ml BMP7 (red) or 100 ng/ml activin A (green) (n = 3, biologically independent samples). Data were expressed as the mean ± S. D. Source data are provided as a Source Data file.

d. Interactions between ALK2 ICD and ActR-IIB ICD in response to ligand stimulation. HEK293A cells were transfected with ActR-IIB-LgBiT and ALK2-SmBiT containing wild-type (left panel) and R206H (right panel) ALK2 as indicated. The cells were stimulated without (blue) or with 100 ng/ml BMP7 (red) or activin A (green). (n = 3, biologically independent samples). Data were expressed as the mean ± S. D. Source data

are provided as a Source Data file.

Suppl. Fig. 6

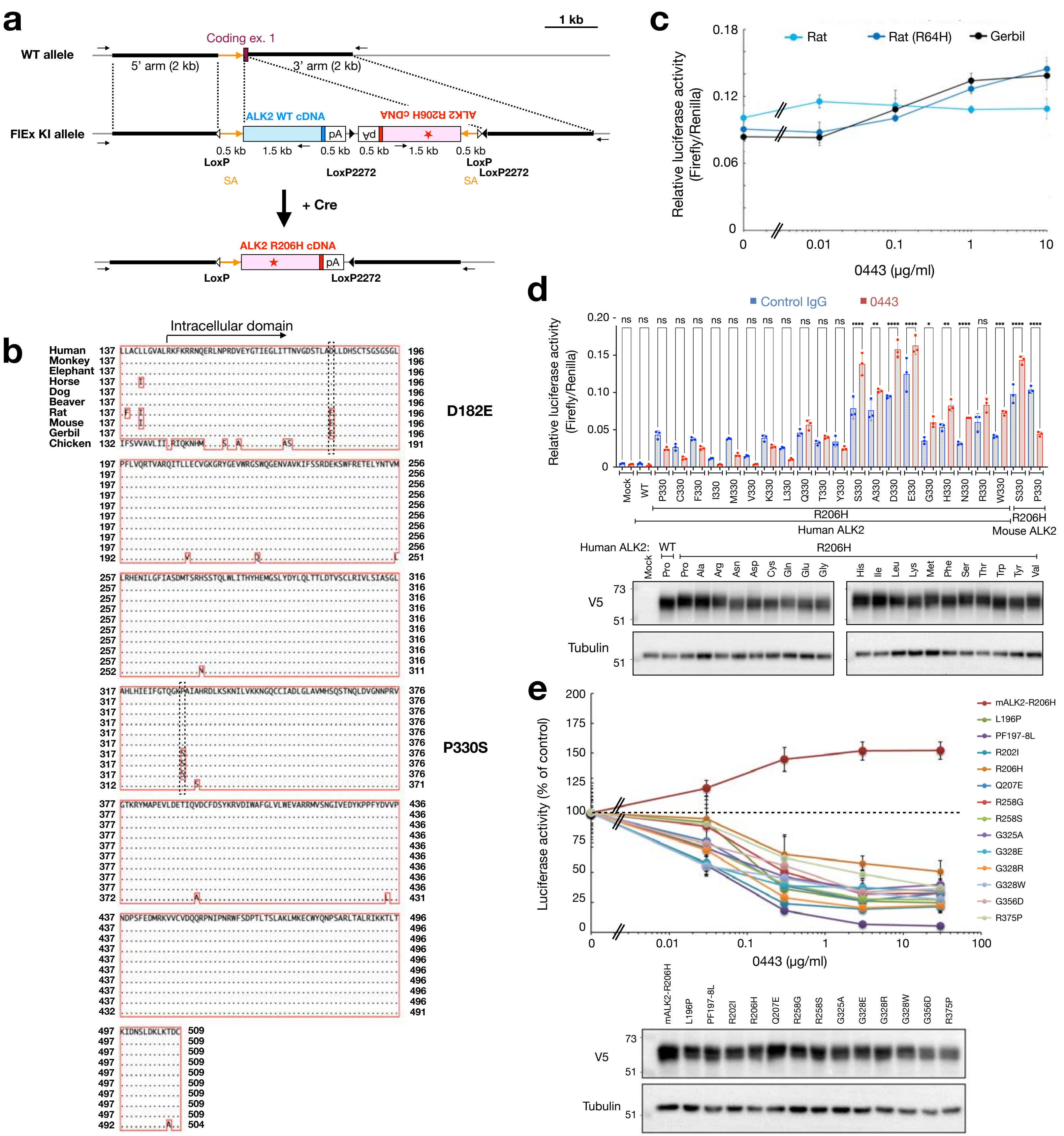
a
WT allele
Coding ex. 1
5' arm (2 kb)
3' arm (2 kb)
1 kb
FIEx KI allele
ALK2 WT cDNA
ALK2 R206H cDNA
LoxP
LoxP2272
SA
+ Cre
b
Intracellular domain
Human
Monkey
Elephant
Horse
Dog
Beaver
Rat
Mouse
Gerbil
Chicken
D182E
P330S
c
Rat
Rat (R64H)
Gerbil
Relative luciferase activity (Firefly/Renilla)
0443 (μg/ml)
d
Control IgG
0443
R206H
Human ALK2
Mouse ALK2
Human ALK2:
V5
Tubulin
e
Luciferase activity (% of control)
0443 (μg/ml)
mALK2-R206H
L196P
PF197-8L
R202I
R206H
Q207E
R258G
R258S
G325A
G328E
G328R
G328W
G356D
R375P

Supplementary Figure 6. Effect of Rm0443 on mouse and human ALK2(R206H).

a. Schematic representation of the mouse Alk2/Acvr1 locus, a targeting vector, and the Alk2/Acvr1 locus structure after Cre-dependent recombination in mouse and human ALK2(R206H) FlEx KI mice. 5’ arm: chr 2: 58,501,090–58,502,971, SA: chr 2: 58,500,551–58,501,089, 3’ arm: chr 2: 58,498,325–58,499,748, 5' CRISPR target: chr 2: 58,501,073–58,501,095, 3' CRISPR target: chr 2: 58,499,743–58,499,765.

b. Alignment of the amino acid sequences of ten animal ALK2 intracellular domains. Amino acid sequences were analyzed using Genetyx Mac ver. 20. Please note that ALK2 sequences from a part of the rodent subfamily, including rats, mice and gerbils, contain two unique conserved amino acid residues, E182 and S330, out of 364 residues.

c. Rm0443 stimulated the R206H mutants of rat ALK2(R64H) and gerbil ALK2 in the absence of ligands. HEK293A cells were transfected with rat ALK2(R206H), rat ALK2(R64H/R206H), or gerbil ALK2(R206H) with reporter plasmids. The cells were stimulated with increasing concentrations of Rm0443. The lowest concentration in the dose response curve represents the absence of Rm0443 (n = 3, biologically independent samples). Data were expressed as the mean ± S. D. Source data are provided as a Source Data file.

d. Effects of Rm0443 on BMP signaling through twenty human ALK2(R206H) constructs carrying a substitution mutation at 330. The P330 residue of hALK2(R206H) was substituted with 19 other amino acid types. Each ALK2(R206H) expression vector was transfected with reporter plasmids in HEK293A cells, and they were cultured with 10 μg/ml control IgG or Rm0443 (n = 3, biologically independent samples). Data were

expressed as the mean ± S. D. The protein levels of hALK2(R206H) were determined by western blot analysis using an antibody against V5-tag. Source data are provided as a Source Data file.

e. Rm0443 suppressed BMP signaling through human ALK2 mutants associated with FOP. HEK293A cells were transfected with one of the twelve types of mutant hALK2 associated with FOP and mALK2(R206H) with reporter plasmids. The cells were treated with increasing concentrations of Rm0443. The lowest concentration in the dose response curve represents the absence of Rm0443 (n = 3, biologically independent samples). Data were expressed as the mean ± S. D. The expression levels of ALK2 were determined by western blotting using an antibody against V5-tag (bottom panels). Source data are provided as a Source Data file.

P values are calculated using unpaired one-way ANOVA and indicated significant if $^*P < 0.05$, $^{**}P < 0.01$, $^{***}P < 0.001$ or $^{****}P < 0.0001$.

Suppl. Fig. 7

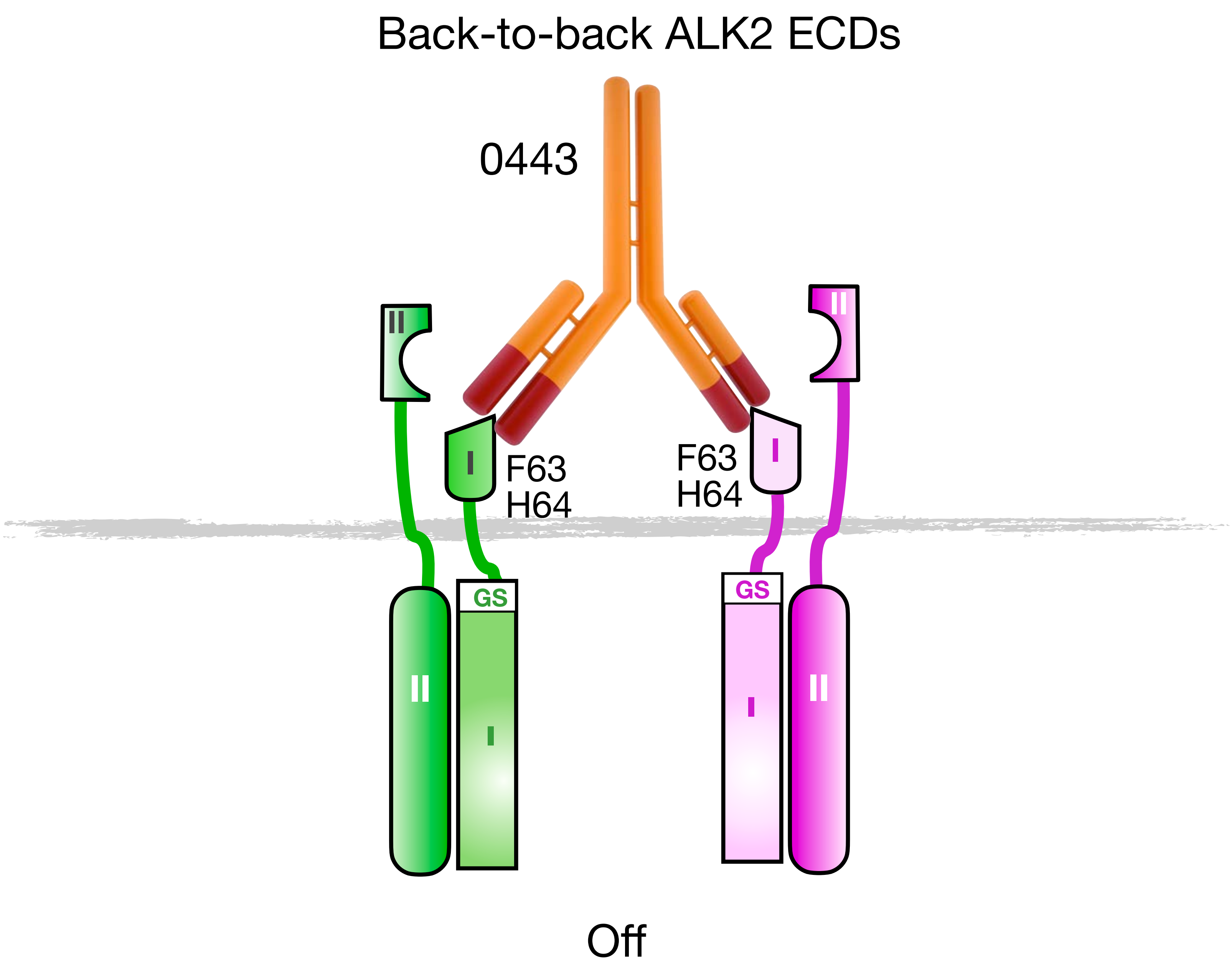
Back-to-back ALK2 ECDs
0443
II
I
F63
H64
F63
H64
I
II
GS
GS
II
I
I
II
Off

Supplementary Figure 7. A schematic model of the inhibition of ALK2 by Rm0443. Rm0443 induces the formation of a unique dimer of ALK2 in a "back-to-back" orientation on the cell membrane by binding residues H64 and F63 on opposite faces of the ligand-binding surface. In this unique conformation of the complex, Rm0443 inhibits the ligand-induced dimer formation of ALK2 ICDs.

Suppl. Fig. 8

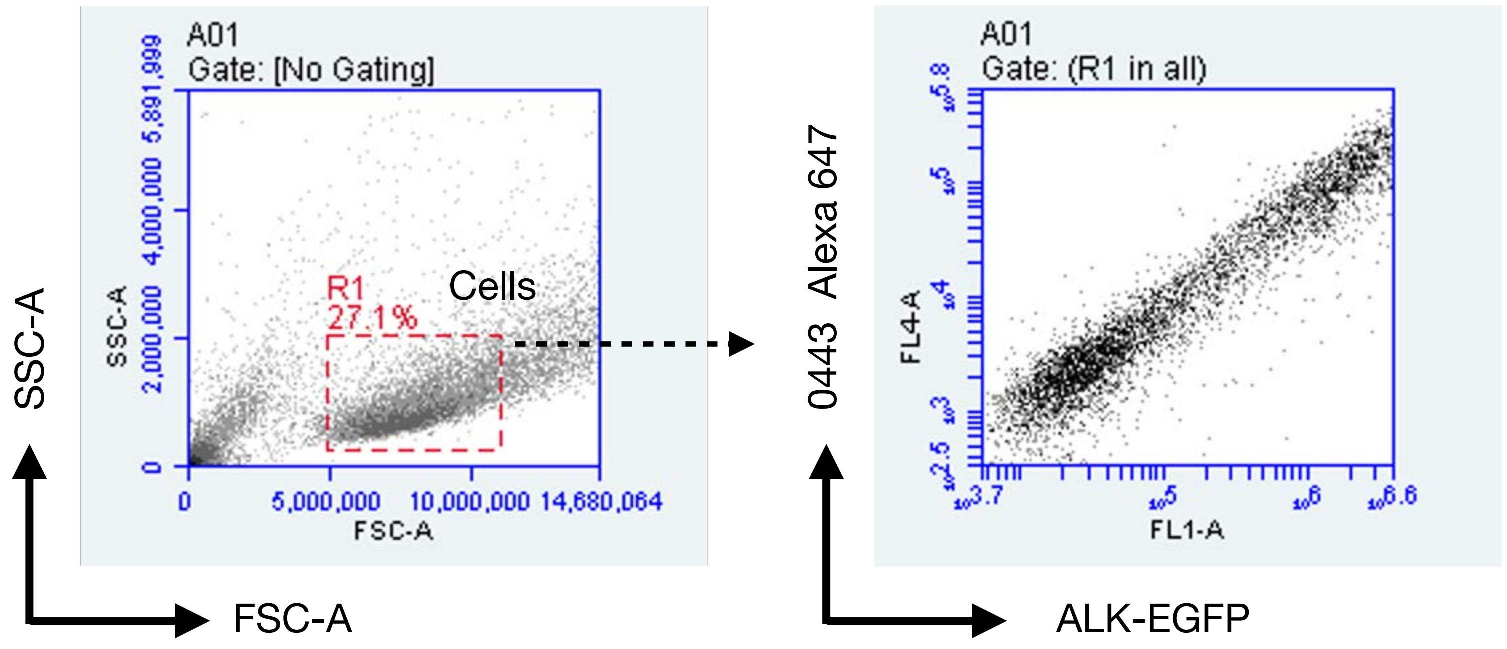

A01
Gate: [No Gating]
R1
27.1%
Cells
SSC-A
FSC-A
0
2,000,000
4,000,000
5,891,999
5,000,000
10,000,000
14,680,064
A01
Gate: (R1 in all)
FL4-A
FL1-A
0443 Alexa 647
ALK-EGFP

Supplementary Figure 8. Exemplifying flow cytometry gating strategy for HEK293A cells. Gating strategy corresponds to flow cytometric analysis in Fig. 1a, Supplementary Fig. 3c and 3d.
