# Supplementary material for: A blocking monoclonal antibody reveals dimerization of intracellular domains of ALK2 associated with genetic disorders
Source: Nat Commun. 2023 May 25;14:2960. doi: 10.1038/s41467-023-38746-5 (PMC10212922; doi:10.1038/s41467-023-38746-5)
Supplement: Supplementary file 2 — Reporting Summary [file 41467_2023_38746_MOESM2_ESM.pdf]

nature portfolio

Corresponding author(s): Takenobu Katagiri

Last updated by author(s): Apr 13, 2023

Reporting Summary

Nature Portfolio wishes to improve the reproducibility of the work that we publish. This form provides structure for consistency and transparency in reporting. For further information on Nature Portfolio policies, see our Editorial Policies and the Editorial Policy Checklist.

Statistics

For all statistical analyses, confirm that the following items are present in the figure legend, table legend, main text, or Methods section.

n/a	Confirmed	
☐	☒	The exact sample size (n) for each experimental group/condition, given as a discrete number and unit of measurement
☐	☒	A statement on whether measurements were taken from distinct samples or whether the same sample was measured repeatedly
☐	☒	The statistical test(s) used AND whether they are one- or two-sided<br>*Only common tests should be described solely by name; describe more complex techniques in the Methods section.*
☒	☐	A description of all covariates tested
☒	☐	A description of any assumptions or corrections, such as tests of normality and adjustment for multiple comparisons
☐	☒	A full description of the statistical parameters including central tendency (e.g. means) or other basic estimates (e.g. regression coefficient) AND variation (e.g. standard deviation) or associated estimates of uncertainty (e.g. confidence intervals)
☐	☒	For null hypothesis testing, the test statistic (e.g. F, t, r) with confidence intervals, effect sizes, degrees of freedom and P value noted<br>*Give P values as exact values whenever suitable.*
☒	☐	For Bayesian analysis, information on the choice of priors and Markov chain Monte Carlo settings
☒	☐	For hierarchical and complex designs, identification of the appropriate level for tests and full reporting of outcomes
☒	☐	Estimates of effect sizes (e.g. Cohen's d, Pearson's r), indicating how they were calculated

Our web collection on statistics for biologists contains articles on many of the points above.

Software and code

Policy information about availability of computer code

Data collection	Flow cytometry data were collected by BD Acuuri ver. 2.2 (BD Biosciences) using C6 software ver. 1.0.264.21 (BD Biosciences).<br>Data for Luciferase reporter assay and Nanoluciferase assay were acquired with the GENios (TECAN) and FLUOstar Omega ver. V5.11 E3 (BMG LabTech).<br>Data for alkaline phosphatase activity were acquired with the Infinite F50 (TECAN).<br>Images of H&E staining, Alcian blue staining and immunohistochemical staining were acquired with the BZ-9000 microscope (Keyence)<br>Bands for Western blotting were visualized with the ChemDock XRS+ system, model no. Universal Hood II (Bio-Rad Laboratories).<br>Heterotopic ossification in mice was scanned using CosmoScan GX ver. GX-1-J-S (Rigaku).<br>X-ray diffraction datasets were collected at beamline BL1A at the Photon Factory (Ibaraki, Japan).<br>Data for binding ability of the antibody to the ALK2 protein was collected by BIAcore T200 (GE Healthcare).
Data analysis	Data analysis and plot were performed using GraphPad Prism 9.<br>Quantitative analysis of the area of heterotopic ossification were performed using Analyze 12.0 software (AnalyzeDirect, Inc.).<br>All molecular graphics were prepared with PyMOL ver. 2.0 (The PyMOL Molecular Graphics System).

For manuscripts utilizing custom algorithms or software that are central to the research but not yet described in published literature, software must be made available to editors and reviewers. We strongly encourage code deposition in a community repository (e.g. GitHub). See the Nature Portfolio guidelines for submitting code & software for further information.

March 2021

Data

Policy information about availability of data

All manuscripts must include a data availability statement. This statement should provide the following information, where applicable:

- Accession codes, unique identifiers, or web links for publicly available datasets
- A description of any restrictions on data availability
- For clinical datasets or third party data, please ensure that the statement adheres to our policy

The coordinates of the refined model has been deposited in the Protein Data Bank (PDB) under the accession code 7RYU[http://doi.org/10.2210/pdb7YRU/pdb]. The PDB codes that are referred in this study are available in the Protein Data Bank under accession codes 2GOO[http://doi.org/10.2210/pdb2GOO/pdb], 1IGT[http://doi.org/10.2210/pdb1IGT/pdb], 3Q4U[http://doi.org/10.2210/pdb3Q4U/pdb], 2QJB[http://doi.org/10.2210/pdb2QJB/pdb], and 3NH7[http://doi.org/10.2210/pdb3NH7/pdb]. All data generated or analysed during this study are included in this published article and its supplementary information files.

Human research participants

Policy information about studies involving human research participants and Sex and Gender in Research.

Reporting on sex and gender	N/A
Population characteristics	N/A
Recruitment	N/A
Ethics oversight	N/A

Note that full information on the approval of the study protocol must also be provided in the manuscript.

Field-specific reporting

Please select the one below that is the best fit for your research. If you are not sure, read the appropriate sections before making your selection.

☒ Life sciences ☐ Behavioural & social sciences ☐ Ecological, evolutionary & environmental sciences

For a reference copy of the document with all sections, see nature.com/documents/nr-reporting-summary-flat.pdf

Life sciences study design

All studies must disclose on these points even when the disclosure is negative.

Sample size	The minimum of samples in each experiment was n=3 and up to n=9. The exact size for each experiment were described in figure legends. The sample sizes were determined based on previous experience. The sample size of in vitro experiment was chosen based on the author's prior experiences with the experiments (Fujimoto et al. (Mol Endocrinol, 2015), Machiya et al. (Bone, 2018), Tsukamoto et al. (Bone, 2020)). Sample sizes for in vivo experiments were chosen based on previous papers in the same research field (Dey et al. (Sci Transl Med, 2015), Lees-Shepard JB et al. (Nat Commun, 2018)).
Data exclusions	No samples or animals were excluded from the analysis.
Replication	At least 2 or 3 replicates were analyzed in each independent experiment to ensure the experimental data were reliable. More than 3 independent experiments were performed to validate key data.
Randomization	The animals and cells were randomly assigned to different experimental groups.
Blinding	For the in vivo experiment, histology analysis and micro-CT analysis were performed and analyzed blindly. For in vitro experiments, the same investigator designed and performed the experiments. Therefore, the investigators were not blinded. For all experiments, colleagues aiding in data collection were blinded. For the in vitro experiment, researchers were not blinded due to the experimental design.

Reporting for specific materials, systems and methods

We require information from authors about some types of materials, experimental systems and methods used in many studies. Here, indicate whether each material, system or method listed is relevant to your study. If you are not sure if a list item applies to your research, read the appropriate section before selecting a response.

Materials & experimental systems

n/a	Involved in the study	
☐	☒	Antibodies
☐	☒	Eukaryotic cell lines
☒	☐	Palaeontology and archaeology
☐	☒	Animals and other organisms
☒	☐	Clinical data
☒	☐	Dual use research of concern

Methods

n/a	Involved in the study	
☒	☐	ChIP-seq
☐	☒	Flow cytometry
☒	☐	MRI-based neuroimaging

Antibodies

Antibodies used	Hybridoma cell lines expressing rat-monoclonal antibodies against ALK2 were established by Integrale Co. (Japan). The anti-ALK2 antibody clone #0443 was purified from the conditioned medium of serum-free Hybridoma-SFM (Thermo Fisher) by affinity chromatography using a protein G column (GE Healthcare) and the Profinia protein purification system (Bio-Rad Laboratories). The concentration of anti-ALK2 antibody for each experiment was described in figure or figure legends.<br>Other antibodies used in this study were as follows.<br>Anti-V5 mouse monoclonal (clone V5005) 1:1,000 immunocytochemistry, Nacalai Tesque, #04434-94.<br>Anti-V5 rabbit monoclonal (clone D3H8Q) 1:1,000 western blot, Cell Signaling Technology, #13202.<br>Anti-α-Tubulin rabbit polyclonal 1:750 western blot, Cell Signaling Technology, #2144.<br>IgG2a isotype control rat monoclonal (clone 54447) in vitro neutralizing assay, R&D, #MAB006.<br>IgG2a isotype control rat monoclonal (clone 2A3) in vivo administration, Bio X Cell, #BE0089.<br>Goat anti-Rat IgG (H+L) Cross-Adsorbed Secondary Antibody, Alexa Fluor 488, in vitro neutralizing assay, Thermo Fisher Scientific, #A11006.<br>Goat anti-Rabbit IgG (H+L) Highly Cross-Adsorbed Secondary Antibody, Alexa Fluor 488 in vitro neutralizing assay, Thermo Fisher Scientific, #A11034.<br>Goat anti-Mouse IgG (H+L) Highly Cross-Adsorbed Secondary Antibody, Alexa Fluor 594, 1:1,000 immunocytochemistry, Thermo Fisher Scientific, #A11032.<br>Goat anti-Rat IgG (H+L) Cross-Adsorbed Secondary Antibody, Alexa Fluor 647, 2 mg/ml Flow cytometry Thermo Fisher Scientific #A21247.<br>Anti-rat IgG, HRP-linked Antibody, 1:1,000 western blot, Cell Signaling Technology #7077.<br>Anti-rabbit IgG, HRP-linked Antibody, 1:1,000 western blot, Cell Signaling Technology #7074.
Validation	The anti-ALK2 rat monoclonal antibody (0443) for immunocytochemistry (Fig. 1a), flow cytometry (Fig. 1a) western blot (Supplemental. Fig. 1c and 1d) and neutralizing assay (Fig. 1c).<br>All listed primary antibodies (anti-mouse and anti-rabbit) except anti-ALK2 for immunocytochemistry and western blot applications were well-established and commercialized products that have been extensively tested and validated by the manufacturers as stated on the websites (Nacalai Tesque, Cell Signaling Technology).

Eukaryotic cell lines

Policy information about cell lines and Sex and Gender in Research

Cell line source(s)	Murine C2C12 cells were obtained from ATCC. Human HEK293A cells and FreeStyle 293F cells were obtained from Thermo Fisher Scientific, respectively.
Authentication	C2C12 cells and HEK293A cells were authenticated by morphology, differentiation capacity and biochemical characteristics in cultures.
Mycoplasma contamination	The cells used in this study were routinely tested for mycoplasma contamination, and they were always negative for it.
Commonly misidentified lines (See ICLAC register)	None of the cell lines used are listed in the database of commonly misidentified cell lines.

Animals and other research organisms

Policy information about studies involving animals; ARRIVE guidelines recommended for reporting animal research, and Sex and Gender in Research

Laboratory animals	C57BL/6 (CLEA Japan Inc, Japan) were used between 9 and 10 weeks. Mouse and human ALK2(R206H) FlEx KI mice in a C57BL/6J background were generated by the Department of Anatomy and Embryology, Faculty of Medicine, University of Tsukuba, and Charles River K. K. Mouse ALK2(R206H) FlEx KI mice were used between 9 and 10 weeks of age. Human ALK2(R206H) FlEx KI mice were crossed with CAG-Cre-ERTg/- (The Jackson Laboratory, USA, stock #004682), and human ALK2(R206H) FlEx KI mice were used at 6 weeks of age. All mice were housed under an SPF condition (12 hour light/dark cycle, 50% relative humidity, and 23 °C) with free access to food and water.
Wild animals	No wild animals were used in the study.

Reporting on sex | The sex of the mice used was described in each figure or figure legend.

Field-collected samples | No field-collected samples were involved in this study.

Ethics oversight | All studies were approved by the Institutional Animal Care and Use Committee and conducted according to the Saitama Medical University Animal Experimentation Regulations (protocol No. 3133).

Note that full information on the approval of the study protocol must also be provided in the manuscript.

Flow Cytometry

Plots

Confirm that:

☒ The axis labels state the marker and fluorochrome used (e.g. CD4-FITC).

☒ The axis scales are clearly visible. Include numbers along axes only for bottom left plot of group (a 'group' is an analysis of identical markers).

☒ All plots are contour plots with outliers or pseudocolor plots.

☒ A numerical value for number of cells or percentage (with statistics) is provided.

Methodology

Sample preparation | Cultured cells were dissociated with an enzyme-free cell dissociation buffer and resuspended in PBS containing 2% FBS. Cells were stained with primary antibodies and secondary antibodies conjugated with fluorochrome according to the manufacturer's protocols.

Instrument | BD Accuri C6 (BD Biosciences).

Software | BD Accuri C6 software (BD Biosciences).

Cell population abundance | All of the experiments used homogeneous cell lines and graph results from 5,000 events per condition.

Gating strategy | FSC vs SSC plot was used to determine cell populations from debris.

☒ Tick this box to confirm that a figure exemplifying the gating strategy is provided in the Supplementary Information.
